# Phosphite-induced changes of the transcriptome and secretome in *Solanum tuberosum* leading to resistance against *Phytophthora infestans*

**DOI:** 10.1186/s12870-014-0254-y

**Published:** 2014-10-01

**Authors:** Dharani Dhar Burra, Oliver Berkowitz, Pete E Hedley, Jenny Morris, Svante Resjö, Fredrik Levander, Erland Liljeroth, Erik Andreasson, Erik Alexandersson

**Affiliations:** Department of Plant Protection Biology, Swedish University of Agricultural Sciences, Alnarp, Sweden; Centre for Phytophthora Science and Management, School of Veterinary and Life Sciences, Murdoch University, Murdoch, WA 6150 Australia; School of Plant Biology, The University of Western Australia, Crawley, WA 6009 Australia; Genome Technology, James Hutton Institute, Invergowrie, Dundee, Scotland; Department of Immunotechnology, Lund University, Lund, Sweden; Present address: Australian Research Council Centre of Excellence in Plant Energy Biology, University of Western Australia, Crawley, WA 6009 Australia

**Keywords:** Phosphite, Late blight, *Phytophthora infestans*, Potato, Secretome, Microarray, Induced resistance, Transgenic lines

## Abstract

**Background:**

Potato late blight caused by the oomycete pathogen *Phytophthora infestans* can lead to immense yield loss. We investigated the transcriptome of *Solanum tubersoum* (cv. Desiree) and characterized the secretome by quantitative proteomics after foliar application of the protective agent phosphite. We also studied the distribution of phosphite *in planta* after application and tested transgenic potato lines with impaired in salicylic and jasmonic acid signaling.

**Results:**

Phosphite had a rapid and transient effect on the transcriptome, with a clear response 3 h after treatment. Strikingly this effect lasted less than 24 h, whereas protection was observed throughout all time points tested. In contrast, 67 secretome proteins predominantly associated with cell-wall processes and defense changed in abundance at 48 h after treatment. Transcripts associated with defense, wounding, and oxidative stress constituted the core of the phosphite response. We also observed changes in primary metabolism and cell wall-related processes. These changes were shown not to be due to phosphate depletion or acidification caused by phosphite treatment. Of the phosphite-regulated transcripts 40% also changed with β-aminobutyric acid (BABA) as an elicitor, while the defence gene PR1 was only up-regulated by BABA. Although phosphite was shown to be distributed *in planta* to parts not directly exposed to phosphite, no protection in leaves without direct foliar application was observed. Furthermore, the analysis of transgenic potato lines indicated that the phosphite-mediated resistance was independent of the plant hormones salicylic and jasmonic acid.

**Conclusions:**

Our study suggests that a rapid phosphite-triggered response is important to confer long-lasting resistance against *P. infestans* and gives molecular understanding of its successful field applications.

**Electronic supplementary material:**

The online version of this article (doi:10.1186/s12870-014-0254-y) contains supplementary material, which is available to authorized users.

## Background

Potato late blight disease caused by the oomycete pathogen *Phytophthora infestans* is among the most severely damaging diseases of the potato crop. The disease is spread by sporangia and zoospores of the oomycete. Under suitable conditions, the encysted zoospores germinate, penetrate the leaf and form haustoria. The initial biotrophic phase leads into a necrotrophic phase characterized by colonization and sporulation on the leaf tissue which in turn gives rise to zoospores for a new cycle of infection [1]. Although there is naturally occurring resistance in wild potato relatives, sustainable resistance has been difficult to achieve at least partly due to rapid adaptation of the oomycete [2,3]. The predominant method to control for late blight disease is through frequent use of fungicides. Because of the overall harmful impact of continual fungicide spray and high costs incurred due to spraying there is a need to develop alternative methods to control late blight disease [4].

One of the alternative methods in pest control that has been pursued in a variety of patho-systems is induced resistance [5,6]. Several reports have shown that plants treated with resistance inducing agents like β-aminobutyric acid (BABA), thiamine (vitamin B1), thiadiazole-7-carbothioc acid S-methyl ester (BTH) and phosphite (H_2_PO_3_^−^) show enhanced resistance after pathogen attack. Recent molecular studies indicate activation of a broad range of defense responses during induced resistance. For example, Ton, et al. [7] showed that BABA-activated defense in Arabidopsis is dependent on a cyclin-dependent kinase–like protein. BABA has also been shown to induce salicylic acid (SA)-dependent induced resistance against *P. infestans* in potato [8,9]. Similarly, metabolically inert phosphite-based compounds that have a direct inhibitory effect on the mycelial growth of *P. infestans* [10-13] have also been shown to induce resistance in potato against late blight disease [14-16]. In agriculture, phosphite-based compounds are marketed as fertilizers, activators of natural resistance or systemic fungicides and are also widely used in some developing countries where they because of the low risk to human health and environment have been identified as potential alternatives to conventional fungicides [17].

However, little is known about the molecular mechanisms behind phosphite-mediated induced resistance and a better understanding of the underlying molecular mechanisms could assist the development of new plant protection strategies. Studies have shown that phosphite enters the cell via phosphate transporters and interferes with phosphate signaling mechanisms because of its close steric resemblance, which potentially could lead to indirect induction of resistance [18-21]. Massoud, et al. [22] demonstrated the importance of the SA pathway in phosphite induced resistance in Arabidopsis against *Hyaloperonospora arabidopsidis*, while Eshraghi, et al. [23] and Machinandiarena, et al. [16] reported that potassium phosphite generates resistance via excessive accumulation of hydrogen peroxide and PR1 expression in Arabidopsis and potato, respectively. In a study of soluble proteins from potato leaf treated with the phosphite product Confine™, Lim, et al. [24] found increased abundance of proteins involved in SA-dependent defense responses, reactive oxygen species (ROS) and calcium-dependent pathways, whereas proteins involved in amino acid and starch metabolism were down-regulated. They also showed induction of hypersensitive response and callose formation after pathogen attack in phosphite treated leaves. This indicates that complex and multiple processes are involved in phosphite-induced resistance.

No previous studies have combined genome-wide transcriptomics with quantitative proteomics on phosphite treated plants. In this study a transcriptomic analysis was complemented by proteomic investigation of secreted proteins in the apoplast, which is regarded as an important interface during plant-pathogen interactions [25]. In parallel, phosphite and phosphate levels were measured in leaves to determine the effect of phosphite on phosphate uptake and metabolism. The level of phosphite-mediated resistance against *P. infestans* was also investigated with transgenic lines deficient in salicylic and jasmonic acid signaling.

## Results

### Phosphite-induced protection against P. infestans is observed in detached leaflet assay throughout the time series

**Figure 1:**
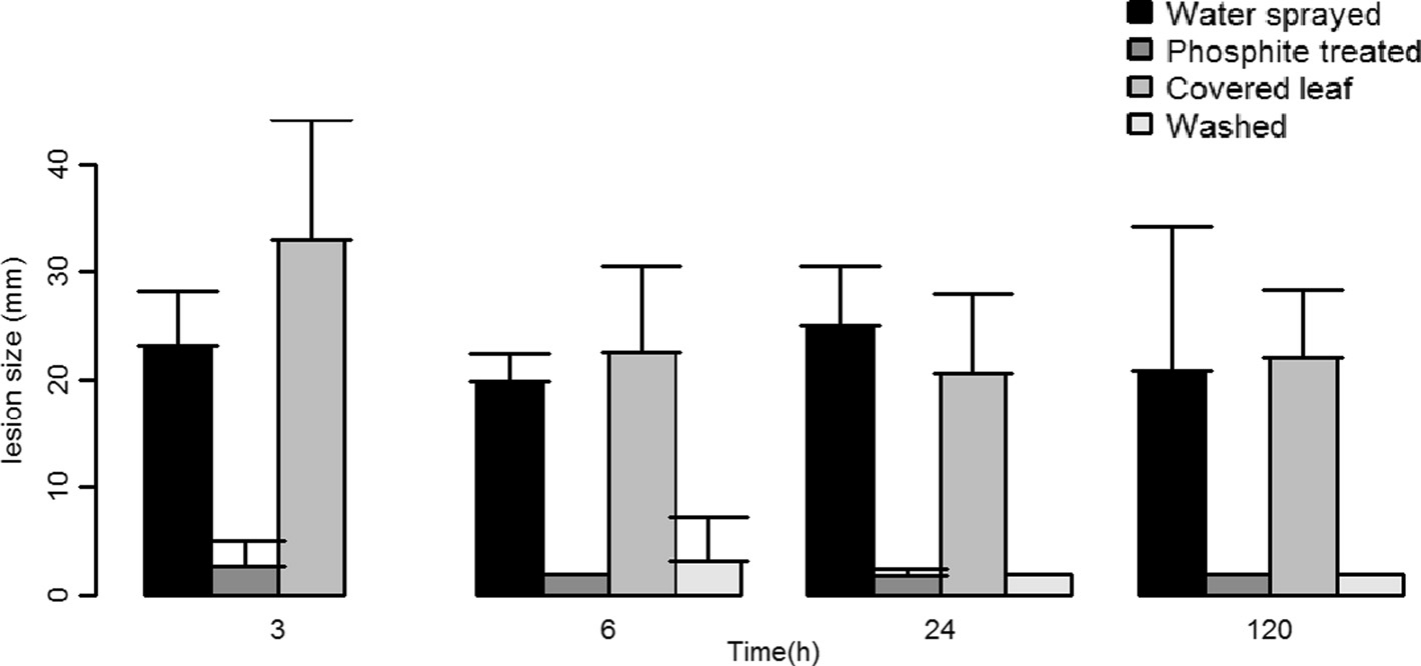
**Detached leaflet assay of potato plants.** Potatoes (cv. Desiree) were foliar sprayed either with 36 mM proalexin (Potassium phosphite; phosphite treated) or tap water (Water treated). “Covered leaves” leaflets were obtained by covering two leaves per plant during phosphite spray. Washed leaflets were obtained by spraying leaves with 36 mM proalexin, washing and drying away the phosphite present on the leaves. Infection was measured as lesion size 7 days after inoculation with *P. infestans*. Data corresponds to mean ± SD obtained from 12 biological replicates.

A clear reduction of *P. infestans* infection was observed at all tested time points after phosphite treatment, i.e. 3, 6, 11, 24 and 120 h (Figure 1). Furthermore, the phosphite treated leaflets showed “HR-like” (“hypersensitive response like”) symptoms at the site of *P. infestans* inoculation compared to extensive sporulation observed on control leaflets 7 days after infection (Additional file 1: Figure S1A). To test whether phosphite was transported in the potato plant and triggered a systemic induced resistance against *P. infestans*, some leaves were covered during treatment. These “covered leaves” were equally infected as the water controls (Figure 1). In order to test if there is a possible direct-effect of phosphite on the pathogen, a set of plants were washed with tap water to remove phosphite from the leaf surface and dried for a minimum of 5 h before sampling for detached leaflet assay. These leaflets, hereafter referred to as “washed”, had similar protection as observed in phosphite treated leaflets (Figure 1).

### Phosphite is distributed rapidly *in planta*

**Figure 2:**
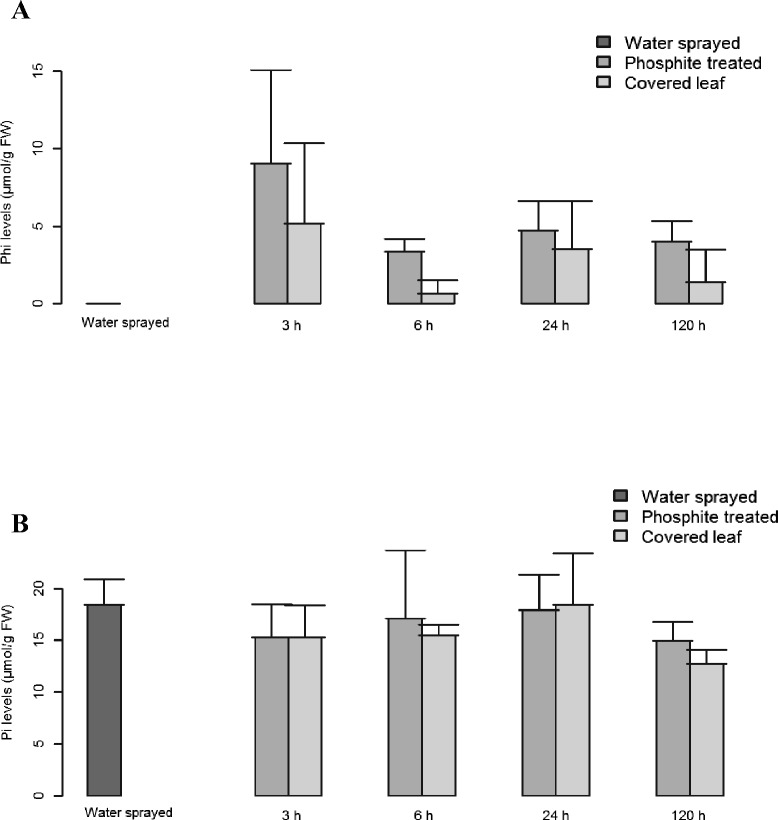
**Phosphite and phosphate measurements.** Phosphite **(A)** and phosphate measurement **(B)** of water sprayed, phosphite treated and “covered leaves” leaflets based on an enzymatic activity assay. Data presented here corresponds to mean ± SD obtained from 3 biological replicates.

An enzymatic assay was used to measure phosphite concentrations in potato leaflets. Accumulation of phosphite was observed in both phosphite treated and “covered leaves” sampled already 3 h after treatment (Figure 2A). Also at 6, 24 and 120 h after treatment, phosphite was detected in both types of leaflets with no significant difference. In order to estimate phosphite levels on the leaf surface, “washed” leaflets were compared to phosphite-treated leaflets. However, no significant difference in phosphite levels were seen between “washed” and “phosphite treated” leaflets (p > 0.05), indicating that phosphite is either taken up completely or it is not possible to remove phosphite from the leaf surface by excessive washing.

No significant difference (p = 0.53) in phosphate levels was detected in phosphite treated leaflets and “covered leaves” leaflets (Figure 2B). In addition, no significant difference (p = 0.19) was observed in phosphate levels of phosphite treated leaflets across time-points. Also phosphate levels did not significantly differ between phosphite treated leaflets and water-sprayed control leaflets (Figure 2B). Similar range of phosphite levels (100–1200 ug/g FW vs. 100–900 ug/g FW by Borza, et al. [26] and phosphate (>1000 ug/g FW) was identified in leaf tissue after Confine™, a phosphite-based fungicide, application on potato when measured by ion chromatography.

### Phosphite treatment has a rapid and transient effect on the leaf transcriptome

**Figure 3:**
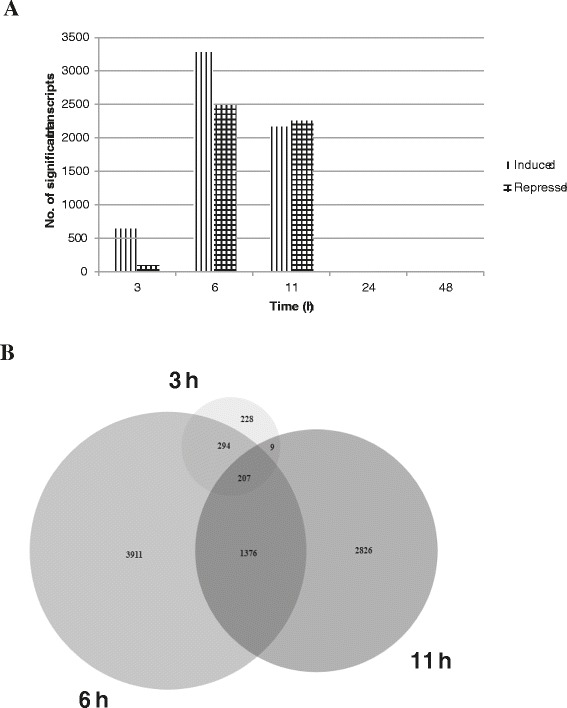
**Differentially expressed genes.** A comparison of number of transcripts induced and repressed at each time point **(A)**, area proportional Venn diagram depicting overlap of transcripts significantly altered at all the time points **(B)**.

Analysis of the microarray data revealed that significant differential expression of transcripts was observed only at 3, 6 and 11 h after phosphite treatment (Figure 3A). In the result section and discussion we only highlight transcripts up- or down-regulated more than two times compared to the control. No transcript was observed to be differentially expressed in leaflets sampled at 24 h, while at 48 h after treatment only one transcript annotated as encoding reticuline oxidase (DMP400036592) was differentially expressed. Since altered pH can effect transcript levels, leaves were sprayed with acidified water (pH 5.2), but no transcript changes were seen at the two time points tested by microarrays, 3 and 11 h after treatment (data not shown). Phosphite has a rapid effect on the leaf transcriptome with 738 transcripts differentially expressed in leaves sampled at 3 h after treatment. The number of differentially expressed transcripts increased to 5788 at 6 h after treatment and decreased to 4418 at 11 h after treatment (Figure 3A). A comparison of the number of up-regulated and repressed transcripts at each time point revealed that 87% of significantly differentially expressed transcripts at 3 h are up-regulated, while this number drops to 57% and 49% at 6 and 11 h, respectively (Figure 3A). A larger overlap of differentially expressed transcripts at time points 6 and 11 h was observed and a “core” of 207 transcripts were differentially expressed at 3, 6 and 11 h (Figure 3B). The expression levels of eight genes were validated by qPCR and a high agreement between microarrays and qPCR was found (R = 0.9; Additional file 2: Figure S2).

### The “core” phosphite-induced transcripts are associated with biotic and abiotic stress responses

**Figure 4:**
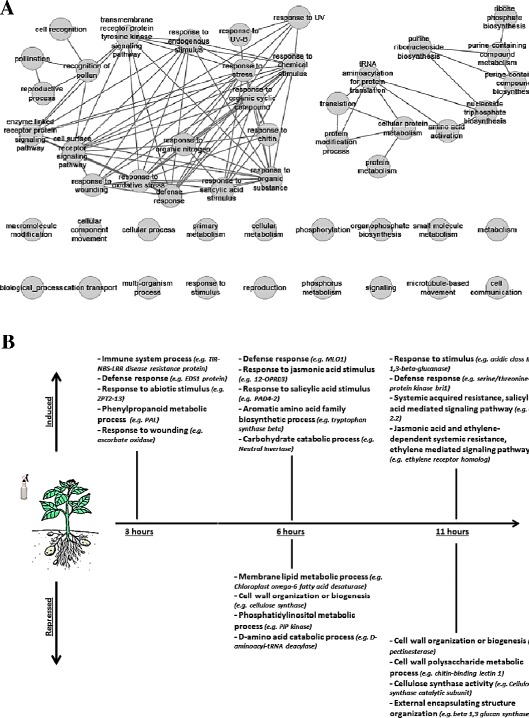
**Gene ontology (GO) analysis.** Clusters of enriched GO terms based on semantic similarity identified among transcripts expressed at all the time points **(A)**, representation of processes and associated example transcripts (in brackets) significantly regulated at each time point **(B)**.

GO analysis of the 207 transcripts significantly up-regulated across all time points identified 174 enriched GO terms. Based on semantic grouping of the enriched GO terms using ReviGO [27], two major clusters were identified (Figure 4A). One cluster contained GO terms such as response to: wounding (GO:0009611), defense (GO:0006952), chitin (GO:0010200), chemical stimulus (GO:0042221) and SA stimulus (GO:0009751). The second cluster was associated with metabolic activity and contained GO terms such as purine nucleotide metabolic process (GO:0006163), nucleoside biosynthetic process (GO:0009163). GO terms that were enriched but did not belong to either of the two clusters were associated with, e.g., phosphate-containing compound metabolic process (GO:0006796) and organophosphate biosynthesis (GO:0019637) (Figure 4A). Transcripts encoding pentacyclic triterpene synthase (PEN1; DMP400036965), Jasmonate ZIM-domain protein 1 (DMP400005281), WRKY transcription factor-30 (DMP400010347), non-race specific disease resistance (NDR1/NHL25;DMP400037182), cytochrome BC1 synthesis protein (BCS1; DMP400036856), wall associated kinase (WAK; DMP400053153) associated with biotic stress were up-regulated across all the time points in addition to transcripts associated with abiotic stress such as ethylene-responsive transcriptional co-activator (DMP400051868), ZPT2-13 (DMP400027219) and salt responsive protein (DMP400000646) (Additional file 3: Table S1).

### Phosphite has a complex effect on stress pathways in addition to affecting processes associated with primary and secondary metabolism

GO analysis of differentially expressed transcripts at 3 h identified enriched GO terms associated with biotic and abiotic stress (Figure 4B, Additional file 4: Figure S3). An increase in expression of defense related transcripts encoding ascorbate oxidase (DMP400009720), cell wall peroxidase (DMP400026523) and phenyl ammonia lyase (PAL; DMP400037388) were observed at this time point. Abiotic stress responsive transcripts encoding salt responsive protein 2 (DMP400000646), early response to dehydration 7 (ERD 7;DMP400010986) associated with dehydration, and transcription factor ZPT2-13 (DMP400027220) were also induced. Transcripts encoding glucan endo-1,3- β -glucosidase (DMP400036382) involved in cell wall biosynthesis and serine/threonine-protein kinase bri1 (DMP400033050) involved in brassinosteroid hormone perception were instead repressed. In addition to activation of defense processes, analysis of differentially expressed transcripts at 6 h revealed significant changes in lipoprotein, amino acid biosynthesis and polysaccharide metabolism (Figure 4B, Additional file 4: Figure S3). Increased expression of a neutral invertase (DMP400023171) transcript involved in sucrose breakdown and phospholipase PLDb1 (DMP400039419) involved in cell wall phospholipid biosynthesis was observed. Significant up-regulation was also observed in genes encoding alternative oxidase (DMP400013470), citrate synthase (DMP400023850), malic enzyme (DMP400004672) and phosphofructokinase (DMP400030437), all of which have been shown to be involved in stress and primary metabolism. Significant up-regulation was also observed in transcripts belonging to amino acid metabolism pathways such as tryptophan synthase (DMP400019982) and chorismate synthase (DMP400042200). Chloroplast omega-6 fatty acid desaturase (DMP400019659) associated with lipid metabolism, phosphatidylinositol-4-phosphate kinase (PIP; DMP400032031) associated with cell wall modification and cellulose synthase (DMP400007078) associated with cell wall biosynthesis were repressed. Analyses of transcripts differentially expressed at 11 h suggest that in addition to activation of stress responses phosphite also has an effect on cell wall related processes (Figure 4B, Additional file 4: Figure S3). Stress related transcripts encoding UDP-Glucosyltransferase (DMP400021191), Glutathione S transferase (DMP400003866), fatty acid desaturase (DMP400041239) and linalool synthase (DMP400048541) were up-regulated. In addition, ontology terms such as response to chemical stimulus (GO:0042221), response to cyclopentenone (GO:0010583) and response to stress (GO:0006950) were enriched. Interestingly, numerous transcripts associated with cell wall related processes were repressed, e.g., pectinesterase (DMP400009250), polygalactouranase (DMP400023907), glycine rich cell wall protein (DMP400050455), chitin binding lectin (DMP400055565) and β-1-3 glucan synthase (DMP400049943). In coherence with this, GO terms such as cell wall modification (GO: 0042545), phosphatidylinositol 3-kinase activity (GO: 0035004) and external encapsulating structure (GO: 0030312) were significantly enriched for the repressed transcripts at 11 h after phosphite treatment (Figure 4B, Additional file 4: Figure S3).

### Phosphite has an effect similar to BABA on the leaf transcriptome

**Figure 5:**
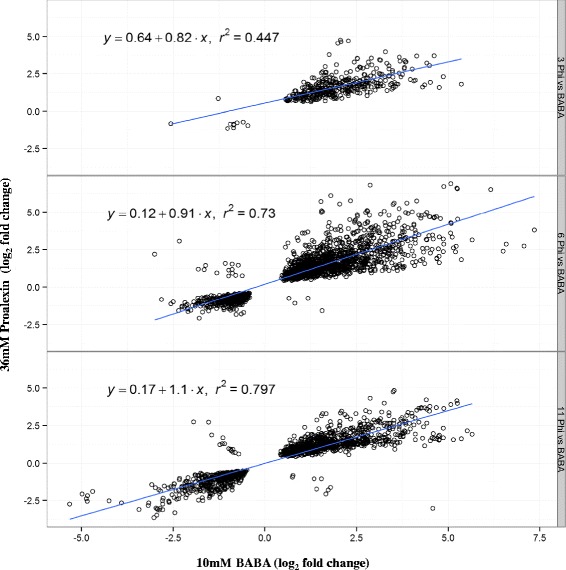
**Phosphite-BABA comparison.** Scatter plots displaying correlation of expression levels of transcripts 48 h after BABA and 3, 6 and 11 h after phosphite (Proalexin) treatment.

A large overlap of differentially expressed genes was observed between phosphite and 48 h after treatment with 10 mM BABA (Figure 5; [28]). Over 300 GO terms were significantly enriched among transcripts that were common to both BABA and phosphite 3 h after treatment (Additional file 5: Table S2). Numerous stress and defense related transcripts such as jasmonate ZIM-domain protein 1 (DMP400005280), thaumatin (DMP400007586), wall-associated kinase (DMP400010164) were common for both treatments. In addition to the large overlap, a high correlation in expression levels of transcripts affected by both BABA and phosphite was observed (Figure 5).

Principal component analysis (PCA) of transcriptomic datasets obtained from microarray experiments with potato clones either susceptible or resistant to *P. infestans* (Ali *et al.* submitted) revealed that the phosphite responsive transcriptome clustered closer to BABA than to uninfected late blight resistant potato clones (cv. Sarpo Mira and cv. SW93-1015) or uninfected susceptible clone (cv. Des; Additional file 6: Figure S4). This suggests that phosphite treatment triggers induced resistance responses on the transcriptomic level that is similar to BABA treatment.

### Secreted proteins involved in cell wall related processes and defense differ in abundance 48 h after treatment

Since no major changes in gene expression was observed from 24 h and onwards, we decided to explore the secretome at a later time point, 48 h, to see whether the marked changes in gene expression were persistent at the protein level. We detected a total of 67 proteins with significant different abundance, whereas only 4 proteins were repressed (Table 1). Mapman visualization showed that proteins associated with cell wall and stress related processes had increased abundance (Additional file 7: Figure S5). Proteins such as ceramidase (DMP400007784), aspartic proteinase nepenthesin-1 (DMP400009572), β-D-glucan exohydrolase (DMP400010541), alpha-galactosidase (DMP400018078), mannose binding lectin (DMP400012540), fasciclin-like arabinogalactan protein 13 (DMP400022582) were observed to change significantly in abundance (Table 1). Significant changes were also observed in stress related proteins such as Kunitz trypsin inhibitor (DMP400046981), peroxidase (DMP400043335), polygalacturonase inhibiting protein (DMP400014905) and Class III peroxidase (DMP400046178). 20 of the proteins identified in this secreted fraction were also observed to change in abundance in the secretome 48 h after BABA treatment [28]. At least 3 proteins encoding chitinases (DMP400002757, DMP400015232 and DMP400015454) were identified in proteins that change in abundance both after phosphite and BABA treatment. However, PR1 induced by BABA was not seen to be induced by phosphite.

**Table 1:** **Secreted proteins changing in abundance 48 h post phosphite treatment**

**PGSC annotation**	**Protein ID**	**Log**_**2**_ **fold change**	**Adjusted p-value**	**SignalP prediction**
Aspartic proteinase nepenthesin-1	DMP400009572	11.6	0.00	Y
Subtilase	DMP400018521	6.7	0.00	N
Beta-hexosaminidase 1	DMP400054227	4.88	0.01	N
Peroxidase N	DMP400041612	4.73	0.04	N
GDSL-lipase protein	DMP400023756	4.07	0.00	N
Subtilase	DMP400058901	3.58	0.02	N
Ceramidase	DMP400007784	3.56	0.00	Y
Major latex	DMP400046294	3.31	0.01	N
Beta-D-glucan exohydrolase	DMP400010541	3.28	0.00	Y
LEXYL2 protein	DMP400051807	3.11	0.00	N
41 kD chloroplast nucleoid DNA binding protein (CND41)	DMP400025990	2.4	0.04	Y
Alpha galactosidase	DMP400018078	2.26	0.01	Y
Peroxidase	DMP400043335	2.22	0.02	Y
Conserved gene of unknown function	DMP400036588	2.12	0.04	N
Class III peroxidase	DMP400046178	2.04	0.04	Y
SBT4C protein	DMP400012124	2	0.04	N
Fasciclin-like arabinogalactan protein 2	DMP400010929	1.74	0.02	Y
Patatin 3	DMP400017707	1.67	0.00	Y
Catechol oxidase B, chloroplastic	DMP400051502	1.65	0.04	N
Kunitz-type protease inhibitor	DMP400016825	1.6	0.03	Y
Fasciclin-like arabinogalactan protein 13	DMP400022582	1.58	0.00	Y
Epidermis-specific secreted glycoprotein EP1	DMP400012540	1.47	0.01	Y
Chitinase	DMP400021005	1.42	0.00	N
Alpha-galactosidase/alpha-n-acetylgalactosaminidase	DMP400020789	1.36	0.02	N
Pectin methylesterase 1	DMP400034073	1.35	0.02	N
Fasciclin-like arabinogalactan protein 14	DMP400037046	1.31	0.04	Y
Polygalacturonase inhibiting protein	DMP400014905	1.27	0.04	Y
Chaperonin-60 beta subunit	DMP400041520	1.24	0.02	N
DUF26 domain-containing protein 2	DMP400030032	1.21	0.03	N
Subtilisin-like protease	DMP400043338	1.19	0.01	Y
Methionine synthase	DMP400015309	1.16	0.00	N
Xylem serine proteinase 1	DMP400033261	1.13	0.00	N
Kunitz trypsin inhibitor	DMP400046980	0.98	0.00	Y
Conserved gene of unknown function	DMP400052225	0.98	0.00	Y
Pathogen-and wound-inducible antifungal protein CBP20	DMP400033771	0.89	0.00	N
Beta-1,3-glucanase, acidic	DMP400014691	0.83	0.02	Y
Kunitz trypsin inhibitor	DMP400046981	0.8	0.00	Y
Class II chitinase	DMP400002757	0.78	0.01	Y
GDSL-lipase 1	DMP400012850	0.74	0.00	Y
Apyrase 3	DMP400012991	0.7	0.03	Y
Pentatricopeptide repeat-containing protein	DMP400005067	0.66	0.04	N
GDSL-like Lipase/Acylhydrolase family protein	DMP400011469	0.64	0.00	N
Basic 30 kDa endochitinase	DMP400015454	0.64	0.00	Y
Serine protease	DMP400007010	0.63	0.01	Y
PAE	DMP400041742	0.6	0.00	Y
Aspartic proteinase nepenthesin-1	DMP400059998	0.6	0.04	Y
Germin	DMP400024701	0.59	0.01	Y
Class III peroxidase	DMP400001015	0.56	0.03	Y
Conserved gene of unknown function	DMP400012143	0.56	0.04	Y
Pectinesterase	DMP400055021	0.56	0.01	N
Endochitinase (Chitinase)	DMP400015232	0.53	0.00	Y
Cucumisin	DMP400010997	0.52	0.00	Y
GDSL-like Lipase/Acylhydrolase family protein	DMP400011470	0.52	0.01	Y
Subtilisin-like protease preproenzyme	DMP400027005	0.52	0.03	Y
STS14 protein	DMP400038079	0.49	0.04	Y
41 kD chloroplast nucleoid DNA binding protein (CND41)	DMP400040088	0.47	0.00	Y
Beta-galactosidase	DMP400014264	0.46	0.01	N
Pectinesterase	DMP400017593	0.45	0.00	Y
Conserved gene of unknown function	DMP400010730	0.42	0.03	Y
Hydrolase	DMP400031772	0.39	0.02	Y
Alpha-glucosidase	DMP400028028	0.37	0.03	N
Reticuline oxidase	DMP400031346	0.36	0.04	Y
Conserved gene of unknown function	DMP400001286	−0.8	0.02	N
Serine carboxypeptidase	DMP400019834	−2.3	0.00	Y
Gene of unknown function	DMP400039337	−2.4	0.02	N
Alpha-galactosidase/alpha-n-acetylgalactosaminidase	DMP400043893	−3.5	0.01	Y
Peptide N-glycanase	DMP400026983	−9.3	0.00	Y

Treatment/control is shown in Log_2_-scale. P-values were adjusted by Benjamini-Hochberg. “Y” and “N” denotes whether the protein was predicted to contain a signal peptide or not by SignalP using default parameters.

### Similar protection observed in phosphite treated jasmonic and salicylic acid potato transgenes

**Figure 6:**
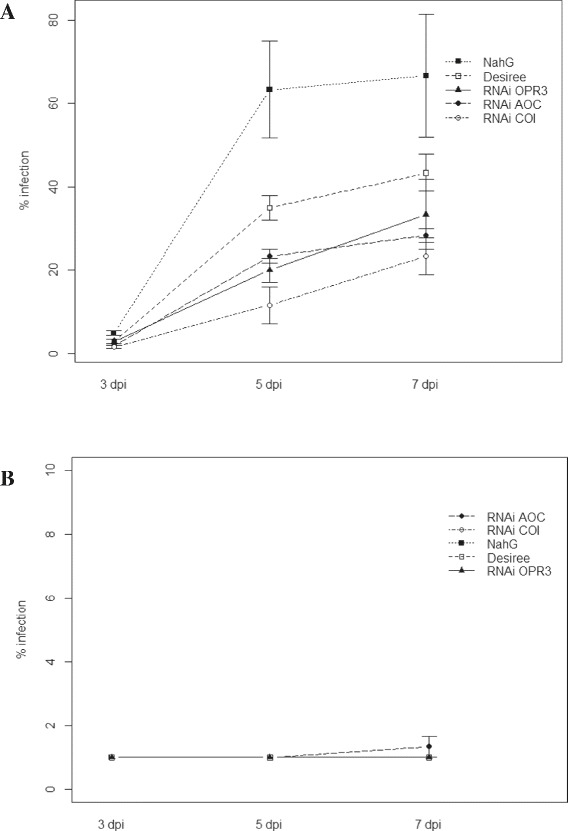
**Whole-plant** ***P. infestans*** **infection.** Progress of disease (% infection) 3, 5 and 7 days post infection in water **(A)** and phosphite treated salicylic acid impaired (NaHG), jasmonic acid impaired knockout (RNAi AOC, RNAi OPR3 and RNAi COIX5) and wild type (cv. Desiree) plants **(B)**. Data presented here corresponds to mean ± SE obtained from 3 biological replicates.

In order to test if phosphite-mediated induced resistance was dependent on SA as shown previously by Massoud, et al. [22] in Arabidopsis, a whole plant infection assay of phosphite and water treated transgenic lines impaired in SA and JA hormone signaling was performed. Analyses of late blight disease symptoms 3, 5 and 7 days post infection revealed that phosphite conferred protection regardless of transgenic line and very few or no symptoms were observed in phosphite-treated transgenic lines in comparison to water treated controls (Figure 6). In untreated plants the NahG line impaired in SA signaling had a lower resistance to *P. infestans* as reported previously (Figure 6A; [29]).

## Discussion

Phosphite provides efficient disease protection in many different plant pathosystems. In the present study we observed resistance against *P. infestans* already at 3 h after phosphite treatment and resistance was sustained even on the 5th day after treatment (Figure 1). The phosphite treatment was accompanied by altered expression of hundreds of transcripts. This study shows for the first time that massive phosphite-induced transcriptomic changes occur rapidly and result in early and sustained protection. Intriguingly, phosphite was detected in parts of treated plants not directly exposed to phosphite already at 3 h after treatment (Figure 2A), demonstrating instantaneous mobility of the molecule inside the plant. Still, phosphite-induced protection was absent in leaves not directly treated with phosphite (Figure 1), suggesting that phosphite treatment leads to local resistance and requires direct foliar application in potato. The *in planta* mobility of phosphite observed is consistent with a recent study by Borza et al. [26], who reported an active translocation of phosphite from leaves to tubers in field-grown potato.

Previous studies have shown that the presence of phosphite impacts phosphate sensing, uptake and metabolism especially when plants are grown in low phosphate conditions [19,21,30-33]. However, in our study no clear differences in phosphate levels were seen within 5 days of phosphite treatment and levels of phosphate were constantly higher than phosphite (Figure 2B) suggesting that phosphite does not change the plant’s phosphate status with our fertilization regime and that this is not the likely cue behind the increase in resistance.

Phosphite is also known to be directly toxic to *P. infestans* [11,13] depending on the concentration used [12,22]. In field applications, the dual nature of the phosphite molecule both being an inducer of plant resistance and having a direct toxic effect on oomycetes might explain the high efficacy. We used the recommended concentration of phosphite that has also resulted in a significant reduction in potato late blight disease in field studies (Liljeroth et al., unpublished data). Although observation of “HR-like” symptoms 7 dpi on phosphite treated leaflets (Additional file 1: Figure S1a), there could still be a direct toxic effect of phosphite. With this in mind, we set out to test if the presence of phosphite on the surface of the leaflets had a direct effect on *P. infestans* infection by washing phosphite-treated leaves before infection. These “washed” leaflets showed the same level of resistance as treated leaflets (Figure 1), indicating that a direct interaction on the leaf surface between *P. infestans* and phosphite was not necessary for resistance. Furthermore, potassium phosphite causes acidification (in our case pH 5.2) and external acidification to pH 4.5 has previously been shown to change the expression of hundreds of genes in *Arabidopsis* [34]. Therefore we tested whether foliar spraying with acidified tap water (pH 5.2) caused an acid-mediated stress leading to increased protection. However, no increased resistance was observed by this treatment (Additional file 1: Figure S1b) and microarray analysis did not show a change in gene expression compared to the control at two time points tested, 3 and 11 h after acidified water treatment. This indicates that phosphite-mediated protection cannot be attributed to its presence on the leaf surface or to its acidifying property. We could also measure phosphite in “covered leaves” without any protection against *P. infestans*, indicating that a strong direct toxic effect is not at play in our experiments.

Machinandiarena, et al. [16] and Massoud, et al. [22] studied a smaller selection of defense related genes first at 72 h after phosphite treatment and did not observe any significant induction of genes then. This is consistent with our observations where we only detected significant changes in the transcriptome at earlier time points with the exception of one transcript at 48 h. In a pre-study, the lack of changes in the transcriptome was confirmed with Aliette, an aluminum-phosphite compound, at 48 h (data not shown). In a proteomic study directed towards soluble proteins, Lim, et al. [24] identified significant changes in abundance of numerous proteins after phosphite treatment in potato. However, after infection of phosphite treated leaves with *P. infestans*, only 4 defense related proteins significantly changed in abundance in comparison to infected control, this led them to hypothesize that phosphite induces pre-activation of defense prior to pathogen inoculation. Indeed, the transcriptomic data analysis presented here reveals that phosphite has an early and transient effect on the transcriptome, which disappears already at 24 h after treatment. Our secretome data also underpins a strong effect of phosphite treatment.

The rapid effect of phosphite 3 h after treatment on the transcriptome with hundreds of transcripts affected includes activation of genes associated to both biotic and abiotic stress response (Figure 4B, Additional file 4: Figure S3). A transcript encoding PAL, a classic marker in plant-pathogen interaction studies associated with SA synthesis, was observed to be up-regulated more than 10-times. BCS1 and NDR1, also associated with SA mediated immunity, were induced as well [35-37]. Of special interest is increased expression of a wall associated kinase (WAK) transcript since WAKs previously have been implicated in mediating defense response during pathogen and wound induced cell-wall damage [38]. Numerous abiotic stress responsive transcripts were also markedly up-regulated. For example, multiple ZPT2-13 transcripts that belong to C2H2 type transcription factor family, which share high sequence homology with Arabidopsis ZAT transcription factors that have been shown to be induced in response to abiotic stress [39].

In addition to activation of stress responses, significant changes in transcripts relating to primary metabolism were observed 6 h after phosphite treatment (Figure 4B, Additional file 4: Figure S3). Reprogramming of primary metabolic processes is one of the hallmarks of plants stress response exemplified by the observed induction of neutral invertases which are known to be rapidly induced during incompatible interactions [40]. Expression of TCA cycle related enzymes such as malic enzyme and citrate synthase also increased, which is an additional indication of heightened defense responses related to metabolism [40]. Fructose bisphosphate aldolase transcript was up-regulated and increased abundance of this protein has been reported in potato leaves after treatment with different inducing agents [41]. On the contrary, Lim, et al. [24] reported decreased abundance of this protein in potato leaves after phosphite treatment by quantitative proteomics. In addition, they observed decreased abundance of sucrose synthase and other proteins related to carbon fixation, carbohydrate metabolism and energy production, whereas we observe an induction of the sucrose synthase transcripts 6 h after phosphite treatment and generally see an up-regulation of transcripts related to these processes. These differences in observations can be due to divergence in transcript and protein regulation, but also due to the differences in experimental set-ups; in the study by Lim, et al. [24] field-grown samples were collected after a series of 5 applications of phosphite 3 days after the final application. The effect of sampling time is also evident in our transcriptomic study as significant repression in processes associated with metabolism were observed 11 h after treatment when compared in samples obtained at 6 h (Additional file 8: Figure S6). In addition we also observed changes on the levels of secreted proteins at 48 h after treatment.

Increased expression of transcripts encoding omega-6 fatty acid desaturases was observed, suggesting heightened defense response after phosphite treatment. These genes have been shown to be repressed during compatible potato-*P. infestans* interactions [42]. Changes in transcripts related to cell wall related processes were also observed (Figure 4B), for example UDP-forming cellulose synthase involved in cellulose biosynthesis. Knock-outs of Arabidopsis homologs of cellulose synthase (IRX5, IRX3 and IRX1) have previously been shown to confer SA, JA and ethylene-independent resistance to *Ralstonia solanaceareum* and *Plectospharella cucmerina* [43]. Previously, Yaeno, et al. [44] demonstrated that avirulence factor Avr3a of *Phytophthora capsici* requires host PIP kinase to stabilize and cause cell death *in planta* in tobacco, suggesting that PIP could be a susceptibility factor and its down-regulation might play a role in early phosphite-mediated induced resistance. Indeed, in this study a PIP kinase associated with various cell wall related processes was repressed both at 6 and 11 h after phosphite treatment.

After 11 h of phosphite treatment a much more pronounced effect on several cell wall modification processes was observed (Figure 4B, Additional file 4: Figure S3). By clustering Arabidopsis microarray data obtained under different stress and chemical treatments, Ma, et al. [45] observed over-representation of cell wall modification processes in clusters with down-regulated genes. Among the cell wall related transcripts, repression of β-1,3-glucan synthase associated to callose biosynthesis. Previous reports have revealed that callose formation can negatively regulate SA-mediated response by physically secluding the site of pathogen penetration [46,47].

Since no major changes in gene expression was observed from 24 h and onwards, we decided to explore the secretome at a later time point to see whether the marked change in gene expression was persistent at the protein level. 63 secreted proteins changed in abundance indicating a prolonged change in protein abundance compared to gene expression. *In silico* functional analysis of these secreted proteins revealed increased abundance of proteins related to cell wall and defense processes (Additional file 7: Figure S5). For example, an aspartic proteinase nepenthesin-1 (Table 1) was found to increase in abundance after phosphite treatment. Numerous secreted aspartic proteases such as constitutive disease resistance 1 (CDR1) have been previously implicated in enhancing resistance in Arabidopsis [48]. A mannose-binding lectin (Table 1) also significantly increased in abundance. These have been shown to play a crucial role during plant pathogen interaction by aiding in recognition of specific glyco-conjugates present on the surface of bacteria and fungi [49]. In addition, a polygalacturonase inhibiting protein, a cell-wall associated protein with well-documented roles in plant defense, and which if over-expressed has been shown to change cell-wall properties prior to pathogen infection, increased in abundance [50,51] further indicating that phosphite effects secreted proteins related to defense and cell wall at later time points. A total of 20 proteins that changed in abundance after phosphite treatment were also found to change after BABA treatment. Proteins encoding chitinases that are members of pathogenesis-related proteins and are produced in response to biotic and abiotic stress [52] were identified among these proteins. This probably indicates the reason behind efficient antifungal activity by phosphite based compounds as mentioned by Deliopoulos, et al. [53] and others since fungal cell walls are primarily composed of chitin.

Massoud, et al. [22] previously demonstrated the importance of the SA pathway in what they described as “priming” when infecting phosphite treated Arabidopsis with the oomycete *Hyaloperonospora arabidopsidis*. Conversely, we observed similar protection on both phosphite-treated transgenic lines impaired in SA and JA signaling, either suggesting that SA and JA act in complementation to mediate induced resistance after phosphite treatment or that the induced resistance response is SA and JA-independent (Figure 6). We observed expression in transcripts associated to both JA- and SA-metabolism and signaling, such as allene oxide synthase and 12-oxophytodienoate reductase 3 both involved in JA biosynthesis and phenylalanine ammonia-lyase and phytoalexin-deficient 4–2 protein involved in SA synthesis and signaling (Additional file 9: Table S3), pointing towards importance of both SA and JA during phosphite-mediated response.

Surprisingly, ~40% overlap among transcripts induced by both phosphite and BABA [28] was observed, a similar trend was also observed from large scale clustering analysis of microarray data, with phosphite effect on the transcriptome clustering closer to BABA than to potato lines resistant to late blight disease (Additional file 6: Figure S4). Interestingly though, we observed significant induction of PR1 after BABA treatment [9], whereas PR1 transcript was not induced after phosphite treatment despite similar protection levels conferred against *P. infestans*.

## Conclusion

This is the first detailed investigation of transcriptomic and proteomic changes after phosphite treated leaves prior to pathogen infection. It seems that multiple defense pathways are rapidly induced by phosphite treatment that causes heightened defense leading to enhanced resistance after pathogen infection in local tissue. Our results also indicate that phosphite treatment influences primary metabolism and cell wall associated metabolic processes, and detailed investigation of these processes, e.g. study of cell wall composition and structure, will deepen the understanding of induced resistance mediated by phosphite. Recently, Arabidopsis plants engineered to use phosphite as their source of phosphate were produced in order to outcompete weeds in a low-phosphate environment [54]. In addition use of phosphite or a combination of phosphite and fungicide has also been shown to be comparatively less expensive fungicides alone in downy mildew disease management in grapevines [55]. A future agricultural system with the relatively inexpensive phosphite as the phosphorus source would have the additional benefit in that phosphite reduces the spread of oomycete and other plant pathogens since it triggers the plant’s innate immune system. It is also evident from research conducted by us and others that there are benefits using phosphite-based salts in various plant protection strategies and improved understanding of induced resistance by phosphite could facilitate expansion of its usage.

## Methods

### Plant material

*Solanum tuberosum* cv. Desiree was grown in 3.5 liter pots in climate chambers for four to five weeks with 16 h light and 8 h dark at 22°C. Plants were foliar sprayed with 40 mL tap water (control) or with 40 mL 1.25% (v/v) Proalexin (LMI AB, Helsingborg, Sweden) diluted in tap water corresponding to 36 mM phosphite as per the recommendation of the manufacturer. Sampling was done at 3, 6, 11, 24, 48 and 120 h. To demonstrate phosphite redistribution in the plant and possible systemic effects on resistance, two leaves of phosphite treated plants were covered with transparent plastic bags to during Proalexin treatment, and are referred to as “covered leaves”. To test whether the acidity of Proalexin could trigger induced resistance, plants were sprayed with acidified tap water adjusted with hydrochloric acid (HCl) to pH 5.4 (equivalent to the pH of the final Proalexin solution). In order to remove Proalexin from the leaf surface, Proalexin-treated plants were washed in tap water and then let to dry for at least 5 h before harvest. Sampling for detached leaflet assay and determination of phosphite levels of so called “washed” leaflets was done for the 6, 11, 24 and 120 h time points. Each treatment was done in three biological replicates at each time point. For the detached leaflet assay, four leaflets from each replicate were sampled while three leaflets were sampled and pooled from each replicate for RNA extraction. Secreted protein fraction or secretome were obtained by pooling five leaflets from each replicate. Six leaflets were pooled from each replicate for phosphate and phosphite measurements.

Transgenic lines [56,57] impaired in salicylic acid (SA) production (NAHGD2) and jasmonic acid (JA) RNAi silenced transgenes of allene oxidase cyclase gene (AOCZ4), 12-Oxophytodienoate reductase 3 gene (OPR3A5, OPR3Z2) and Coronatine insensitive gene (COIX5) were cultivated the same way as the control (cv. Desiree).

### *P. infestans* infection assay

*P. infestans* strain 88069 (kindly provided by Francine Grovers, Wageningen University) was used for detached leaflet and whole plant infection assays performed as in Ali, et al. [58]. For the detached leaf assay two 20 μl (15,000 zoospores/ml) drops were spotted on the abaxial side of the leaflet. Lesion size for the detached leaflet assay was measured seven days post inoculation. For the whole plant infection assay comparing phosphite treated transgenic lines and background control (cv. Desiree), the plants were either sprayed with tap water or 36 mM Proalexin, and 24 h after treatment plants were sprayed with a solution containing 15,000 zoospores/ml. Percentage infection was scored visually as percentage of infected leaf area.

### Phosphite and phosphate measurements

For the 3, 6, 24 and 120 h time points, phosphate and phosphite were extracted from approximately 40 mg freeze-dried leaf tissue with 1% (v/v) acetic acid at a 1:10 mass:volume ratio using a tissue lyser (Qiagen Australia Pty. Ltd., Doncaster, Australia) for 1 min at 25 Hz. After clearing the lysate by centrifugation at 14,000 *g* and 4°C for 15 min, the supernatant was assayed for phosphate and phosphite as described [59,60]. Statistically significant differences between phosphite and phosphate levels was determined using ANOVA (general linear model) with leaflet sample type (phosphite treated/“covered leaves”/ “washed”) and time-point as fixed factors in Minitab 16.

### RNA extraction, microarray analysis and verification by qPCR

RNA was isolated with Qiagen RNeasy mini kits (Qiagen, Hilden, Germany) according to the manufacturer’s instructions. RNA quality was determined on a Nanodrop spectrophotometer (Thermo, Saveen Werner, Malmö, Sweden) and integrity was tested on a BioRad Experion (BioRad, Herecules, CA) before further analysis. For mRNA expression analysis a custom-made expression array (Agilent JHI *Solanum tuberosum* 60 k v1) based on the predicted transcripts in the *Solanum phureja* genome (version 3.4) was run according to the supplier’s (Agilent) instructions. The complete microarray design is available in ArrayExpress (A-MEXP-2272).

The resulting probe intensities were background corrected and normalised using the quantile method in the Limma R-package (Smyth, Gordon K. 2004). Fold changes and standard errors were obtained by fitting a linear model to each gene and standard errors were smoothed by empirical Bayes. Genes with p < 0.05 (Benjamini-Hochberg adjusted) were regarded as statistically significant. The microarray data was deposited in the ArrayExpress database (accession number E-MTAB-2243). Venn diagrams were drawn using BioVenn [61]. Gene ontology (GO) terms for probes were constructed by clustering 26 plant genomes using a parallelized version of OrthoMCL according [28]. GO term enrichment was performed using GOEast [62] and gene ontology (GO) terms with p < 0.05 (Benjamini-Yekutieli adjusted) were regarded as significantly enriched, clusters from significantly enriched GO term lists were created in ReviGO [27] with default semantic cluster settings (SimRel allowed similarity = 0.7). Cytoscape (v 2.8.3) [63] was used for visualization of GO term clusters. Using Qlucore Omics explorer v 2.2 (Qlucore AB, Lund, Sweden) with variance filter adjusted to 0.25, an unsupervised principal component analysis of gene expression data produced with the Agilent JHI Solanum tuberosum 60 k v1 microarray from 10 mM BABA after 48 h (cv. Desiree; [28]), uninfected late blight resistant clones (cv. Sarpo Mira and SW93-1015), uninfected late blight susceptible cultivar (cv. Desiree) [64] was performed. Expression levels of significantly differentially expressed transcripts 48 h after 10 mM BABA treatment and 3, 6 and 11 h after Proalexin treatment were compared using a linear regression model in ggplot2 in R.

The expression levels of eight genes (StPEN1, StAOS, StFAD3, StMLO1, StWRKY8, StLX-3R, StWRKY1, StNOD084) were validated by qPCR. Primers were designed with the help of Primer 3 [65] according to criteria which include a predicted melting temperature of 57-62°C, a primer length of 18–24 nucleotides, a product size of 100–250 base pairs (bp) and a GC content of 30–70%. Primer sequences are given in Additional file 10: Table S4. For cDNA synthesis 500 ng of total RNA was transcribed to cDNA using SuperScript® III Reverse Transcriptase including degradation with RNase H according to the manufacturer’s protocol (InvitroGen). qPCR was performed with a CFX96 (ABI) using Power SYBR® Master Mix (InvitroGen) and PCR cycles ran according to the manufacturer’s recommendations. The comparative CT method was used for relative quantification of transcripts [66]. A high agreement between microarrays and qPCR was found (R = 0.9; Additional file 8: Figure S6).

### Secretome isolation and mass spectrometry of secreted proteins

Secretome isolation was performed using vacuum infiltration with phosphate buffer saline according to a previously described protocol [58]. The secreted protein fraction was dissolved in 6x SDS-PAGE buffer containing DTT, and denatured at 95°C for 3 minutes. Each of the pooled samples (30 μl) was loaded on polyacrylamide gels and separated for 2 cm with SDS-PAGE. After staining with Coomassie, the gel lane from each sample was cut into about 1 mm^2^ pieces. Samples were then subjected to in-gel tryptic digestion with incubation (modified sequencing grade; Promega, Madison, WI, USA) overnight at 37°C. Peptides were extracted in 50–80% acetonitrile and excess acetonitrile was vapourised using centrifugation under vacuum. De-salting was performed using UltraMicro spin columns (Nest group).

LC-MS/MS analysis was performed on a LTQ Orbitrap XL ETD with an Eksigent nano-LC system (Eksigent technologies, Dublin, CA, USA). A 5 μl sample was injected and separated at a flow rate of 300 nl/min with a 90 minute gradient. The four most intense ions were selected in data-dependent mode and fragmented in the linear ion trap, with settings as in Ali, et al. [58]. Files were converted to mzML [67] and Mascot Generic Format (MGF) using ProteoWizard [68] and uploaded to the Proteios Software Environment, ProSE [69]. MGF files were used for MS/MS identification, and mzML files for feature detection using msInspect [70]. Peptide and protein identification were performed in Mascot (http://www.matrixscience.com) and X!Tandem (http://thegpm.org/tandem/) in a database consisting of all Solanum proteins in UniProt (http://www.uniprot.org) and all annotated proteins from the potato genome project [71], extended with an equal amount of decoy (reverse sequence) proteins for false discovery rate (FDR) estimation. The MS mass tolerance was set to 5 ppm and MS/MS fragment tolerance to 0.5 Da, with one potential missed cleavage allowed. Cysteine carbamidomethylation was set as fixed and methionine oxidation as variable modification. Peptide cutoff for the combined searched were set to an FDR rate of 1% as described previously [72] within ProSE [69]. Label-free quantification of peptides was performed using a precursor intensity-based strategy [73]. To quantify possible peptides, msInspect [70] feature detection was performed from ProSE using default settings. The features were matched to MS/MS identifications with a retention time tolerance of 0.2 minutes and an m/z tolerance of 0.005 Da as well as a requirement of same charge and LC-MS/MS run. Alignment of peptide features between LC-MS/MS runs was performed within ProSE using the built-in algorithm described by Sandin, et al. [74]. A report of the features corresponding between runs was exported for further analysis. The proteomics data was deposited in PRIDE with the project accession: PXD001031.

### Secretome data analysis

Peptides with a FDR of < 0.01 were selected for further analysis. For normalization, we used the Eigen MS method incorporated in DanteR (v0.2) that uses Eigenvalues to find trends in the data for normalization [75,76]. In DanteR, data was filtered, missing values imputed and an ANOVA was run on the peptide level as before. After Benjamini-Hochberg adjustment, differentially expressed peptides with p < 0.05 were selected for further analysis. The median fold-change of peptides associated to the same proteins was calculated. Functional analysis of identified proteins was performed using MapMan [77]. Signal sequence was predicted with SignalP 4.1 [78] using default parameters.

### Availability of supporting data

The microarray data was deposited in ArrayExpress, accession number: E-MTAB-2243. The proteomics data was deposited in PRIDE with the project accession: PXD001031.

## Additional files

Additional file 1: Figure S1. (A) “HR like” symptoms observed at the site of *P. infestans* infestation in phosphite treated leaflets (top panel) while extensive sporulation of *P. Infestans* observed on water treated leaflets (bottom panel) 7 dpi in the detached leaflet assay, (B) detached leaflet assay of Water sprayed (control) and acidified water sprayed leaflets. Additional file 2: Figure S2. Correlation between microarrays and qPCR for eight different genes specified in Additional file 10: Table S4. Additional file 3: Table S1. The. List of “core” of phosphite induced transcripts that are differentially expressed at 3, 6 and 11 h post treatment. Additional file 4: Figure S3. Selection of significantly enriched gene ontology terms at each time point (Benjamini-Yekutieli adj. p-value <0.05). Additional file 5: Table S2. List of significantly enriched gene ontology terms among transcripts regulated both by BABA 48 h after treatment and phosphite 3 h after treatment. Additional file 6: Figure S4. Principal component analysis of transcriptomic data obtained from 3 biological replicates of BABA treated plants (cv. Desiree, 10 mM BABA 48 h post treatment) referred to as BABA, phosphite treated plants (cv. Desiree, 36 mM phosphite) sampled 3 (Phi3), 6 (Phi6) and 11 (Phi11) h after phosphite treatment, uninfected late blight resistant clones [58] cv Sarpo Mira (SMC) and clone SW93-1015 (SWC) and uninfected late blight susceptible clone cv. Desiree (DesC). Additional file 7: Figure S5. Functional analysis using MapMan of secreted proteins with significantly changed abundance detected 48 h after phosphite treatment [77]. Additional file 8: Figure S6. Mapman visualization of metabolic processes in transcripts significantly changing 6 and 11 h after treatment. Additional file 9: Table S3. Significant differences observed in transcripts associated with salicylic and jasmonic acid pathways, the markers were selected as suggested in Studham, et al. [37]. Additional file 10: Table S4. Primer sequences for qPCR.

## Abbreviations

BABA: β-aminobutyric acid
BTH: S-methyl ester
SA: Salicylic acid
JA: Jasmonic acid
ROS: Reactive oxygen species
“HR-like”: “Hypersensitive response like”
FW: Fresh weight
PEN1: Pentacyclic triterpene synthase
NDR1/NHL25: Non-race specific disease resistance
BCS1: Cytochrome BC1 synthesis protein
PAL: Phenyl ammonia lyase
ERD7: Early response to dehydration 7
PIP: Phosphatidylinositol-4-phosphate
PCA: Principal component analysis
PR1: Pathogenesis responsive protein 1
AOC: Allene oxidase cyclase
OPR3: 12-Oxophytodienoate reductase
COI: Coronatine insensitive
GFP: Green fluorescent protein
ANOVA: Analysis of Variance
SDS-PAGE: Sodium dodecyl sulphate - Polyacrylamide gel electrophoresis
LTQ: Linear trap quadropole
MS: Mass spectrometry
LC: Liquid chromatography
dpi: Days post infection
GO: Gene ontology
