# Supplementary material for: Phosphite-induced changes of the transcriptome and secretome in Solanum tuberosum leading to resistance against Phytophthora infestans
Source: BMC Plant Biol. 2014 Oct 1;14:254. doi: 10.1186/s12870-014-0254-y (PMC4192290; doi:10.1186/s12870-014-0254-y)
Supplement: Additional file 1: Figure S1. — (A) “HR like” symptoms observed at the site of P. infestans infestation in phosphite treated leaflets (top panel) while extensive sporulation of P. Infestans observed on water treated leaflets (bottom panel) 7 dpi in the detached leaflet assay, (B) detached leaflet assay of Water sprayed (control) and acidified water sprayed leaflets. [file 12870_2014_254_MOESM1_ESM.pptx]

## Slide 1
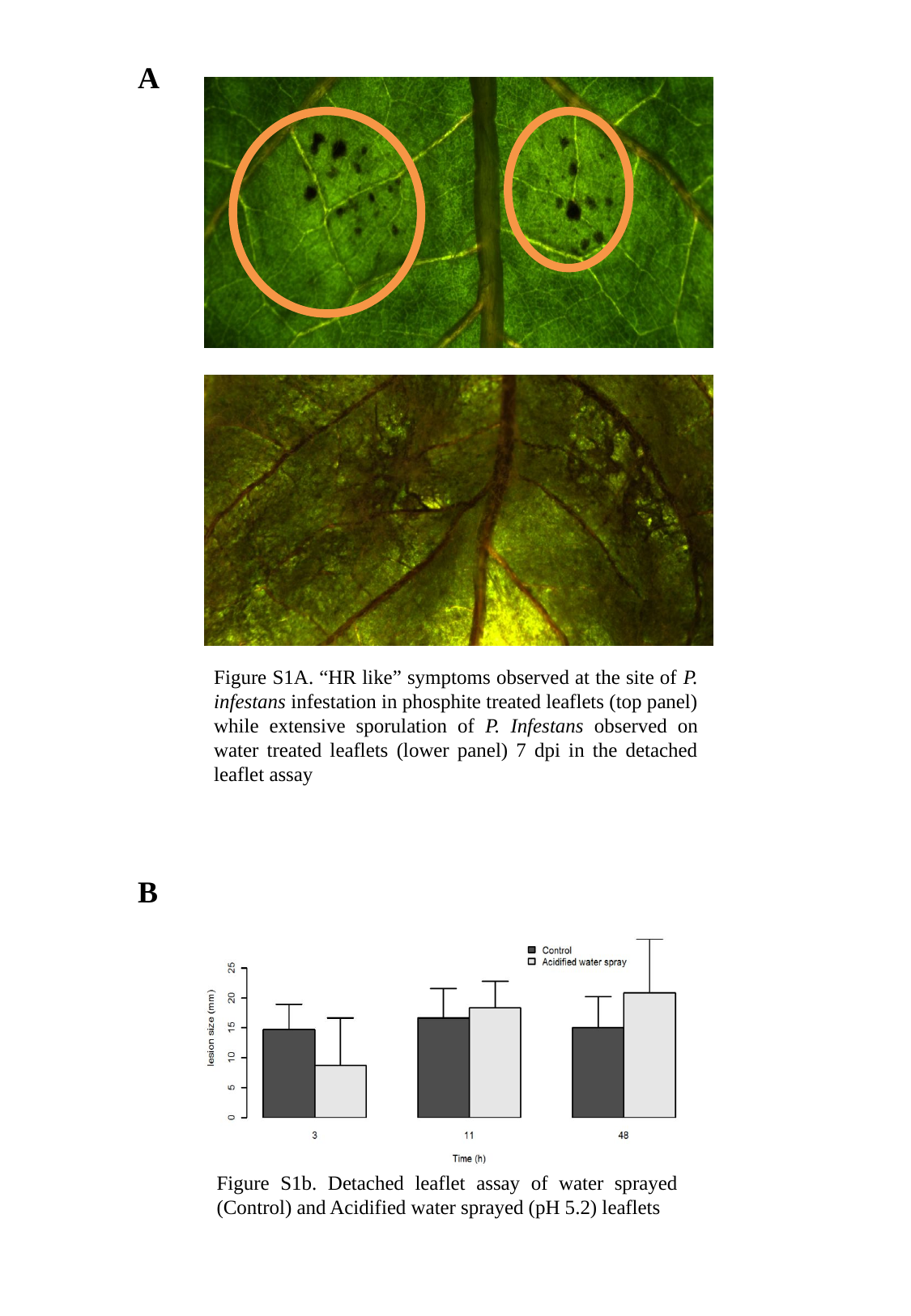

A
Figure S1A. “HR like” symptoms observed at the site of P. infestans infestation in phosphite treated leaflets (top panel) while extensive sporulation of P. Infestans observed on water treated leaflets (lower panel) 7 dpi in the detached leaflet assay
B
Figure S1b. Detached leaflet assay of water sprayed (Control) and Acidified water sprayed (pH 5.2) leaflets
