# Supplementary material for: Phosphite-induced changes of the transcriptome and secretome in Solanum tuberosum leading to resistance against Phytophthora infestans
Source: BMC Plant Biol. 2014 Oct 1;14:254. doi: 10.1186/s12870-014-0254-y (PMC4192290; doi:10.1186/s12870-014-0254-y)
Supplement: Additional file 2: Figure S2. — Correlation between microarrays and qPCR for eight different genes specified in Additional file 10: Table S4. [file 12870_2014_254_MOESM2_ESM.docx]

Figure S2. Correlation between microarrays and qPCR for eight different genes specified in Table S4.
