# Supplementary material for: Phosphite-induced changes of the transcriptome and secretome in Solanum tuberosum leading to resistance against Phytophthora infestans
Source: BMC Plant Biol. 2014 Oct 1;14:254. doi: 10.1186/s12870-014-0254-y (PMC4192290; doi:10.1186/s12870-014-0254-y)
Supplement: Additional file 3: Table S1. — The. List of “core” of phosphite induced transcripts that are differentially expressed at 3, 6 and 11 h post treatment. [file 12870_2014_254_MOESM3_ESM.docx]

Supplementary Table 1. The core of phosphite induced transcripts - List of significant transcripts (Benjamini-Hochberg adj. p-value <0.05) that are differentially expressed at 3, 6 and 11 hours post treatment.

| **ID** | **3 hours log_2_ fold change** | **6 hours log_2_ fold change** | **11 hours log_2_ fold change** | **Putative function** |
| --- | --- | --- | --- | --- |
| DMP400055668 | 2.06 | 3.53 | 1.43 | Conserved gene of unknown function |
| DMP400003898 | 0.9 | 0.68 | 0.85 | Conserved gene of unknown function |
| DMP400047791 | 1.11 | 1.45 | 1.49 | Serine-threonine protein kinase, plant-type |
| DMP400047773 | 1.35 | 1.76 | 1.12 | Serine-threonine protein kinase, plant-type |
| DMP400021459 | 1.43 | 1.96 | 1.04 | N-rich protein |
| DMP400002390 | 2.89 | 3.29 | 2.57 | Calcium ion binding protein |
| DMP400053153 | 1.08 | 1.32 | 0.88 | Wall-associated kinase |
| DMP400007305 | 1.56 | 3.08 | 1.39 | DNA binding protein |
| DMP400007304 | 1.5 | 3.12 | 1.48 | DNA binding protein |
| DMP400042782 | 1.36 | 1.46 | 1.06 | Rubber elongation factor protein |
| DMP400017844 | 1.88 | 3.66 | 2.52 | Geraniol 10-hydroxylase |
| DMP400042615 | 0.88 | 1.27 | 1.16 | UDP-glucosyltransferase |
| DMP400042691 | 1.68 | 2.82 | 1.88 | Brassinosteroid LRR receptor kinase |
| DMP400042614 | 0.91 | 1.38 | 1.16 | UDP-glucosyltransferase |
| DMP400042613 | 0.92 | 1.06 | 0.85 | UDP-glucosyltransferase |
| DMP400005281 | 2.19 | 3.01 | 1.79 | Jasmonate ZIM-domain protein 1 |
| DMP400005280 | 2.21 | 3.11 | 1.91 | Jasmonate ZIM-domain protein 1 |
| DMP400017845 | 1.68 | 3.66 | 2.41 | Geraniol 10-hydroxylase |
| DMP400012010 | 4.26 | 3.06 | 2.13 | Conserved gene of unknown function |
| DMP400012399 | 2.11 | 2.8 | 1.36 | PRIB5 protein |
| DMP400031369 | 1.98 | 1.79 | 1.38 | Cyclic nucleotide-gated calmodulin-binding ion channel |
| DMP400044883 | 2.52 | 5.23 | 2.58 | UDP-glucose:glucosyltransferase |
| DMP400024487 | 1.17 | 2.18 | 1.33 | S-locus-specific glycoprotein S6 |
| DMP400061074 | 2.64 | 5.76 | 2.92 | Conserved gene of unknown function |
| DMP400047003 | 1.08 | 1.82 | 1.07 | Protein transport protein SEC61 subunit gamma |
| DMP400046972 | 2.26 | 2.74 | 1.57 | DC1 domain containing protein |
| DMP400015429 | 1.71 | 1.8 | 1.36 | Conserved gene of unknown function |
| DMP400009557 | 2.93 | 4.13 | 2.69 | Sigma factor binding protein 1 |
| DMP400036965 | 1.8 | 2.89 | 1.33 | PEN1 |
| DMP400030290 | 1.95 | 2.44 | 1.27 | Heat shock factor protein |
| DMP400030243 | 2.12 | 2.69 | 1.94 | ATFP4 |
| DMP400030198 | 1.39 | 1.52 | 1.2 | Receptor-like kinase |
| DMP400030255 | 0.85 | 0.77 | 0.69 | Receptor-like kinase |
| DMP400030189 | 1.86 | 2.18 | 1.33 | Receptor-like kinase |
| DMP400030196 | 1.64 | 1.13 | 1.12 | Receptor-like kinase |
| DMP400030195 | 1.7 | 1.45 | 1.35 | Receptor-like kinase |
| DMP400013331 | 2.35 | 2.47 | 1.5 | Conserved gene of unknown function |
| DMP400059655 | 1.32 | 1.53 | 1.26 | Hcr2-0A |
| DMP400033298 | 1.55 | 1.69 | 1.08 | ATP binding protein |
| DMP400048938 | 0.8 | 2.03 | 0.62 | Calmodulin binding protein |
| DMP400009760 | 1.76 | 2.57 | 2.65 | Hcr2-0A |
| DMP400008389 | 1.67 | 2.53 | 2.68 | Hcr2-0A |
| DMP400019456 | 3.38 | 4.85 | 2.96 | Soul heme-binding family protein |
| DMP400013823 | 2.37 | 1.05 | 1.51 | RNA-binding protein |
| DMP400013958 | 0.93 | 0.83 | 1.18 | Conserved gene of unknown function |
| DMP400037182 | 1.92 | 2.27 | 1.48 | NHL25 (NDR1/HIN1-LIKE 25) |
| DMP400029580 | 1.38 | 2.01 | 1.77 | Conserved gene of unknown function |
| DMP400019452 | 1.86 | 4.27 | 1.84 | Glutathione-S-transferase |
| DMP400052917 | 2.63 | 4.11 | 2.25 | Heat shock cognate 70 kDa protein 1 |
| DMP400019451 | 1.83 | 4.4 | 1.91 | Glutathione-S-transferase |
| DMP400014356 | 1.28 | 1.84 | 1.37 | Calcium-binding allergen Ole e |
| DMP400051967 | 1.33 | 2.27 | 1.66 | Glucosyltransferase |
| DMP400018208 | 3.67 | 4.61 | 1.62 | Ethylene-responsive transcription factor |
| DMP400007094 | 1.16 | 1.14 | 0.73 | ATP binding protein |
| DMP400007096 | 2.04 | 1.52 | 0.97 | ATP binding protein |
| DMP400043768 | 1.92 | 2.89 | 1.23 | Ankyrin repeat-containing protein |
| DMP400016299 | 1.1 | 2.05 | 1.31 | Leucine-rich repeat family protein |
| DMP400016297 | 1.2 | 2.01 | 1.32 | Leucine-rich repeat family protein |
| DMP400050563 | 1.44 | 1.3 | 1.23 | Conserved gene of unknown function |
| DMP400014395 | 1.33 | 2.08 | 1.66 | WRKY transcription factor |
| DMP400051868 | 1.53 | 3.87 | 1.34 | Ethylene-responsive transcriptional coactivator |
| DMP400022294 | 1.4 | 2.22 | 1.06 | LIM domain containing protein |
| DMP400022256 | 1.42 | 3.1 | 1.59 | GTP cyclohydrolase II / 3,4-dihydroxy-2-butanone-4-phosphate synthase |
| DMP400022279 | 0.96 | 0.76 | 0.72 | Protein phosphatase 2c |
| DMP400024286 | 1.09 | 1.18 | 0.9 | BED finger-nbs-lrr resistance protein |
| DMP400036620 | 1.63 | 3.37 | 2.15 | Hsr203J |
| DMP400010347 | 2.77 | 3.05 | 1.33 | WRKY transcription factor-30 |
| DMP400010348 | 2.94 | 2.69 | 1.71 | WRKY transcription factor-30 |
| DMP400028410 | 1.72 | 0.92 | 1.09 | Conserved gene of unknown function |
| DMP400000707 | 1.1 | 2.06 | 1.27 | Serine-threonine protein kinase, plant-type |
| DMP400000646 | 3.54 | 2.73 | 1.82 | Salt responsive protein 2 |
| DMP400018009 | 1.2 | 1.44 | 0.74 | Avr9/Cf-9 rapidly elicited protein 140 |
| DMP400009535 | 2.1 | 2.73 | 2.26 | Calmodulin-binding protein |
| DMP400030956 | 1.2 | 1.04 | 1.03 | Receptor protein kinase |
| DMP400030957 | 1.28 | 1.03 | 1.07 | Receptor protein kinase |
| DMP400055433 | 1.54 | 1.5 | 0.83 | NDR1 |
| DMP400055306 | 1.7 | 1.65 | 0.9 | NDR1 |
| DMP400005849 | 2.94 | 3.47 | 1.45 | Conserved gene of unknown function |
| DMP400037388 | 3.73 | 3.51 | 1.29 | Phenylalanine ammonia-lyase |
| DMP400055761 | 0.88 | 1.57 | 0.75 | Enhanced disease susceptibility 1 protein |
| DMP400022623 | 0.76 | 1.32 | 0.98 | Calmodulin |
| DMP400014135 | 1.57 | 1.07 | 0.94 | Hydrolase |
| DMP400000533 | 2.04 | 2.23 | 1.17 | Conserved gene of unknown function |
| DMP400032151 | 0.86 | 1.12 | 1.13 | Serine-threonine protein kinase, plant-type |
| DMP400032135 | 1.89 | 2.2 | 1.31 | Gene of unknown function |
| DMP400000379 | 0.88 | 0.68 | 0.68 | Conserved gene of unknown function |
| DMP400008459 | 1.42 | 2.56 | 1.28 | GEM 5 |
| DMP400008437 | 1.28 | 1.69 | 1.45 | Glucosyltransferase |
| DMP400012850 | 2.07 | 2.05 | 1.7 | GDSL-lipase 1 |
| DMP400012851 | 2.18 | 2.26 | 1.68 | GDSL-lipase 1 |
| DMP400025802 | 1.05 | 1.43 | 1 | Nucellin |
| DMP400025803 | 1.07 | 1.5 | 1.05 | Nucellin |
| DMP400048303 | 3.25 | 3.37 | 2.37 | Conserved gene of unknown function |
| DMP400041617 | 0.81 | 1.27 | 0.69 | Nematode resistance |
| DMP400041616 | 1.08 | 1.31 | 0.77 | Nematode resistance |
| DMP400056119 | 2.41 | 2.3 | 1.34 | Cf-2.2 |
| DMP400045798 | 1.87 | 2.15 | 1.16 | Serine/threonine protein kinase family protein |
| DMP400045799 | 2.04 | 2.69 | 1.18 | Serine/threonine protein kinase family protein |
| DMP400045795 | 1.53 | 2.06 | 1.04 | Gamma-glutamyl-gamma-aminobutyrate hydrolase |
| DMP400045801 | 1.91 | 2.49 | 1.17 | Serine/threonine protein kinase family protein |
| DMP400045800 | 2.26 | 2.99 | 1.39 | Serine/threonine protein kinase family protein |
| DMP400008627 | 2.5 | 2.46 | 1.46 | Auxin-regulated dual specificity cytosolic kinase |
| DMP400008626 | 2.44 | 2.32 | 1.24 | Auxin-regulated dual specificity cytosolic kinase |
| DMP400038123 | 3.94 | 4.09 | 1.48 | DNA binding protein |
| DMP400011864 | 2.04 | 2.82 | 1.68 | CXE carboxylesterase |
| DMP400011863 | 2.78 | 5 | 3.11 | HSR203J protein |
| DMP400037406 | 2.38 | 2.77 | 1.97 | 21kD protein |
| DMP400043281 | 0.85 | 0.96 | 1.07 | Sigma factor sigb regulation protein rsbq |
| DMP400036214 | 1.21 | 1.16 | 0.91 | Serine-threonine protein kinase, plant-type |
| DMP400036232 | 1.12 | 1.57 | 0.91 | Serine-threonine protein kinase, plant-type |
| DMP400053863 | 3.01 | 4.79 | 3.15 | Cucumber peeling cupredoxin |
| DMP400043259 | 1.47 | 2.96 | 1.28 | Cytoplasmic protein of eukaryotic origin (38.3 kD) |
| DMP400043258 | 1.5 | 2.75 | 1.48 | Cytoplasmic protein of eukaryotic origin (38.3 kD) |
| DMP400016944 | 2.28 | 2.86 | 1.69 | Zinc finger protein PIF1 |
| DMP400011221 | 1.69 | 2.1 | 1.72 | Conserved gene of unknown function |
| DMP400054257 | 1.99 | 2.87 | 2.23 | WRKY transcription factor |
| DMP400045491 | 1.49 | 1.59 | 1.53 | Conserved gene of unknown function |
| DMP400045489 | 1.43 | 1.7 | 1.6 | Conserved gene of unknown function |
| DMP400028022 | 1.01 | 1.88 | 1.06 | Conserved gene of unknown function |
| DMP400055617 | 1.6 | 3.17 | 1.76 | NAC domain protein |
| DMP400055618 | 1.59 | 3.34 | 1.71 | NAC domain protein |
| DMP400053436 | 1.54 | 1.72 | 0.78 | Arogenate dehydrogenase |
| DMP400009493 | 1.04 | 1.75 | 1.05 | Calreticulin |
| DMP400055104 | 1.8 | 3.26 | 2.76 | Hexose transporter |
| DMP400036856 | 3.51 | 4.82 | 1.43 | BCS1 protein |
| DMP400036855 | 3.49 | 4.73 | 1.45 | BCS1 protein |
| DMP400045417 | 3.11 | 3.7 | 1.69 | MYB domain class transcription factor |
| DMP400045384 | 1.94 | 2.79 | 1.35 | S-locus-specific glycoprotein S6 |
| DMP400059528 | 0.68 | 0.83 | 0.84 | Ornithine cyclodeaminase |
| DMP400010640 | 2.25 | 1.72 | 1.47 | Conserved gene of unknown function |
| DMP400047535 | 2.01 | 4.1 | 2.53 | UDP-glucose:glucosyltransferase |
| DMP400020631 | 3.19 | 3.71 | 2.39 | WRKY-type transcription factor |
| DMP400055482 | 1.99 | 4 | 2.33 | N-acetyltransferase |
| DMP400055483 | 3.53 | 3.94 | 1.88 | N-acetyltransferase |
| DMP400055481 | 1.86 | 3.08 | 1.33 | N-acetyltransferase |
| DMP400045913 | 1.98 | 3.07 | 1.57 | Serine-threonine protein kinase, plant-type |
| DMP400037342 | 1.02 | 2.44 | 1.54 | CYP81B36 |
| DMP400039840 | 1.88 | 1.91 | 1.29 | EIX receptor 1 |
| DMP400018259 | 1.05 | 0.77 | 0.86 | Leucyl-tRNA synthetase |
| DMP400035312 | 3.66 | 5.74 | 3.52 | Prephenate dehydrogenase |
| DMP400010905 | 1.21 | 2.03 | 1.24 | Short-chain alcohol dehydrogenase |
| DMP400022416 | 1.83 | 1.61 | 1.25 | Conserved gene of unknown function |
| DMP400003432 | 2.16 | 2.7 | 1.6 | Matrix metalloprotease 1 |
| DMP400003431 | 2.19 | 2.65 | 1.5 | Matrix metalloprotease 1 |
| DMP400027219 | 4.86 | 5.18 | 3.34 | ZPT2-13 |
| DMP400027217 | 4.73 | 5.35 | 3.7 | ZPT2-13 |
| DMP400027220 | 4.49 | 5.77 | 2.83 | ZPT2-13 |
| DMP400027271 | 4.89 | 5.25 | 3.04 | ZPT2-13 |
| DMP400026278 | 2.05 | 3.18 | 1.92 | VQ motif-containing protein |
| DMP400017125 | 1.26 | 1.65 | 1.05 | Guanylate kinase |
| DMP400063324 | 2.3 | 3.2 | 2.32 | DUF26 domain-containing protein 1 |
| DMP400009719 | 3.25 | 3.2 | 2.69 | Ascorbate oxidase |
| DMP400009720 | 1.86 | 2.79 | 1.4 | Ascorbate oxidase |
| DMP400007586 | 1.39 | 1.56 | 1.19 | Thaumatin |
| DMP400001237 | 1.74 | 1.55 | 1.14 | Stellacyanin CASLP1 |
| DMP400033934 | 1.87 | 2.7 | 2.52 | Calcium ion binding protein |
| DMP400032500 | 1.52 | 1.98 | 1.51 | Amino acid transporter |
| DMP400025936 | 1.74 | 3.63 | 2.42 | Hcr2-0A |
| DMP400007947 | 1.25 | 1.25 | 0.83 | Conserved gene of unknown function |
| DMP400050606 | 3.42 | 3.08 | 1.62 | Aromatic amino acid decarboxylase 1B |
| DMP400050613 | 1.91 | 1.54 | 1.23 | Aromatic amino acid decarboxylase 1B |
| DMP400009690 | 0.84 | 1.55 | 0.78 | Multifunctional protein |
| DMP400009691 | 1.04 | 1.97 | 0.83 | Multifunctional protein |
| DMP400011351 | 0.91 | 1 | 0.73 | Receptor protein kinase |
| DMP400018457 | 2.81 | 2.77 | 1.73 | CHP-rich zinc finger protein |
| DMP400026001 | 3.8 | 4.79 | 1.21 | N-hydroxycinnamoyl-CoA:tyramine N-hydroxycinnamoyl transferase THT7-1 |
| DMP400025990 | 1.22 | 1.27 | 1.24 | 41 kD chloroplast nucleoid DNA binding protein (CND41) |
| DMP400025999 | 3.58 | 4.58 | 2.41 | Tyramine hydroxycinnamoyl transferase |
| DMP400001406 | 1.09 | 1.43 | 0.7 | Glucan endo-1,3-beta-glucosidase |
| DMP400053996 | 1.51 | 1.92 | 1.12 | Nucleoredoxin |
| DMP400053995 | 1.51 | 1.93 | 1.1 | Nucleoredoxin |
| DMP400014552 | 1.15 | 1.17 | 0.81 | LRR receptor-like serine/threonine-protein kinase FLS2 |
| DMP400013232 | 1.31 | 1.36 | 0.54 | Phospholipid-transporting ATPase 9 |
| DMP400014817 | 0.93 | 1.11 | 0.72 | SCL domain class transcription factor |
| DMP400029304 | 1.22 | 1.32 | 1.12 | Double WRKY type transfactor |
| DMP400043818 | 2.81 | 3.67 | 1.99 | Pentatricopeptide repeat-containing protein |
| DMP400043851 | 2.62 | 4.67 | 1.77 | Conserved gene of unknown function |
| DMP400029336 | 1.97 | 2.37 | 2.35 | Avr9/Cf-9 rapidly elicited protein 75 |
| DMP400014887 | 1.6 | 2.57 | 1.65 | ATP binding protein |
| DMP400048541 | 3.28 | 3.3 | 2.79 | Ascorbate oxidase |
| DMP400048542 | 3.37 | 3.25 | 2.75 | Ascorbate oxidase |
| DMP400048540 | 3.33 | 3.34 | 2.62 | Ascorbate oxidase |
| DMP400050458 | 2.38 | 1.92 | 2.18 | Glycine-rich cell wall structural protein 1 |
| DMP400001976 | 1.99 | 1.85 | 1.37 | Serine/threonine-protein kinase PBS1 |
| DMP400044133 | 1.57 | 1.62 | 1.57 | Gene of unknown function |
| DMP400044130 | 2.42 | 2.9 | 2.01 | Conserved gene of unknown function |
| DMP400041757 | 1.79 | 1.81 | 1.84 | Serine-threonine protein kinase, plant-type |
| DMP400018962 | 2.43 | 3.79 | 3.02 | ERF transcription factor 4 |
| DMP400023205 | 1.63 | 1.39 | 1.08 | DNA-binding protein |
| DMP400011757 | 1.1 | 0.77 | 1.15 | Hcr9-OR2A |
| DMP400026523 | 4.53 | 3.84 | 1.67 | Cell wall peroxidase |
| DMP400017179 | 1.9 | 3.06 | 1.2 | Conserved gene of unknown function |
| DMP400003867 | 1.84 | 2.5 | 1.04 | Glutathione-S-transferase |
| DMP400065467 | 3.2 | 4.98 | 3.57 | UDP-glucose:glucosyltransferase |
| DMP400028068 | 3.03 | 4.69 | 3.46 | Glucosyl/glucuronosyl transferases |
| DMP400034718 | 1.18 | 1.37 | 1.69 | Conserved gene of unknown function |
| DMP400015660 | 3.25 | 3.6 | 2.16 | Conserved gene of unknown function |
| DMP400041966 | 1.25 | 1.11 | 1.48 | Receptor protein kinase zmpk1 |
| DMP400041968 | 1.24 | 1.14 | 1.24 | Receptor protein kinase zmpk1 |
| DMP400015622 | 1.8 | 1.83 | 1.61 | DC1 domain-containing protein |
| DMP400009947 | 1.37 | 1.36 | 1.26 | Calmodulin-binding protein |
| DMP400017469 | 1.2 | 2.17 | 1 | Ubiquitin-protein ligase |
| DMP400051086 | 2.9 | 4.05 | 2.14 | TSI-1 protein |
| DMP400004578 | 1.71 | 4.21 | 2.34 | Coatomer subunit beta'-2 |
| DMP400039687 | 1.26 | 1.42 | 1.23 | Salt responsive protein 1 |
| DMP400006063 | 2.07 | 4.27 | 2.43 | Amino acid transporter |
| DMP400006062 | 2.29 | 4.41 | 2.48 | Amino acid transporter |
