# Supplementary figures and images for: Phosphite-induced changes of the transcriptome and secretome in Solanum tuberosum leading to resistance against Phytophthora infestans
Source: BMC Plant Biol. 2014 Oct 1;14:254. doi: 10.1186/s12870-014-0254-y (PMC4192290; doi:10.1186/s12870-014-0254-y)

## Slide 1
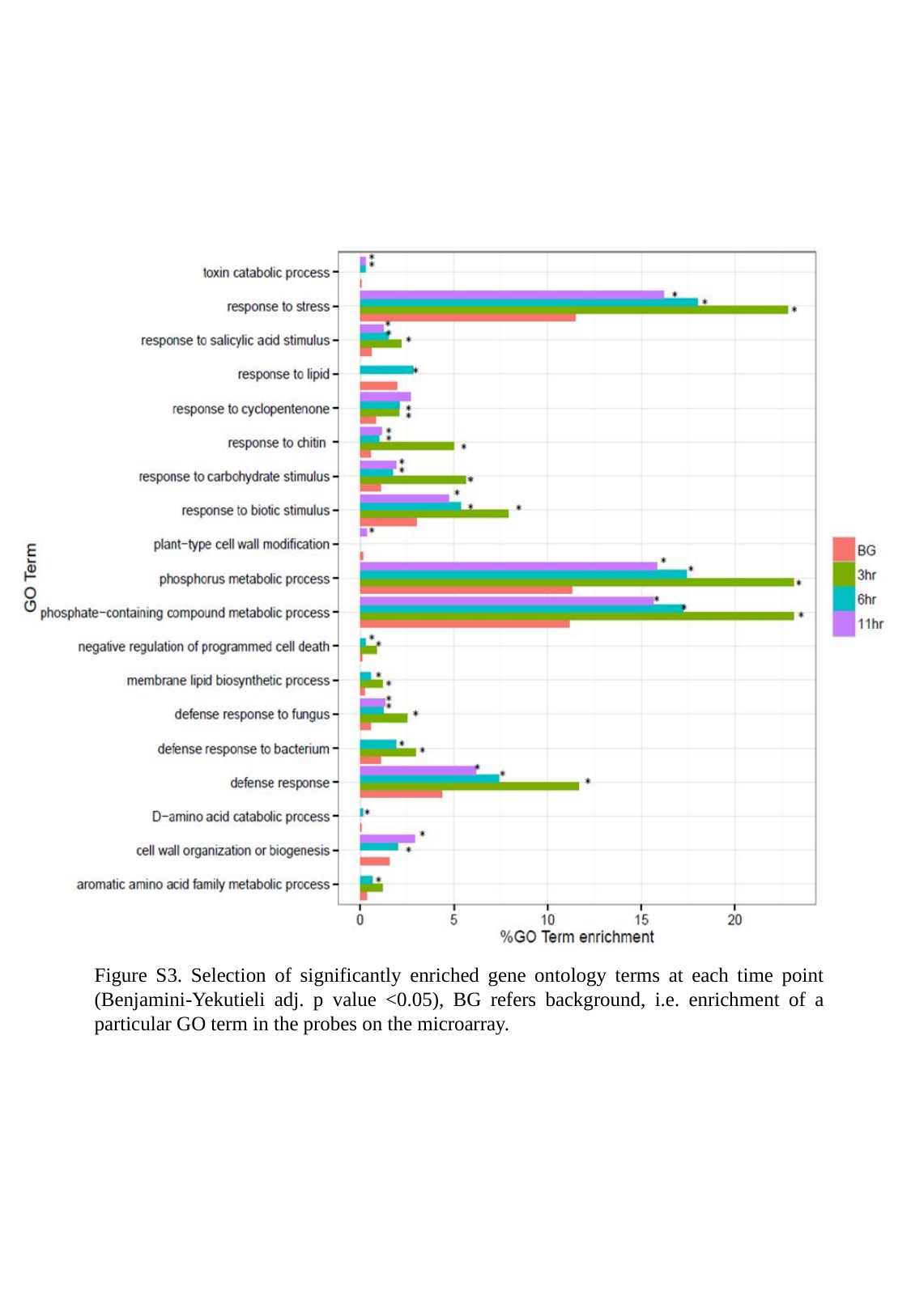

Supplement: Additional file 4: Figure S3. — Selection of significantly enriched gene ontology terms at each time point (Benjamini-Yekutieli adj. p-value <0.05). [file 12870_2014_254_MOESM4_ESM.pptx]

## Slide 1
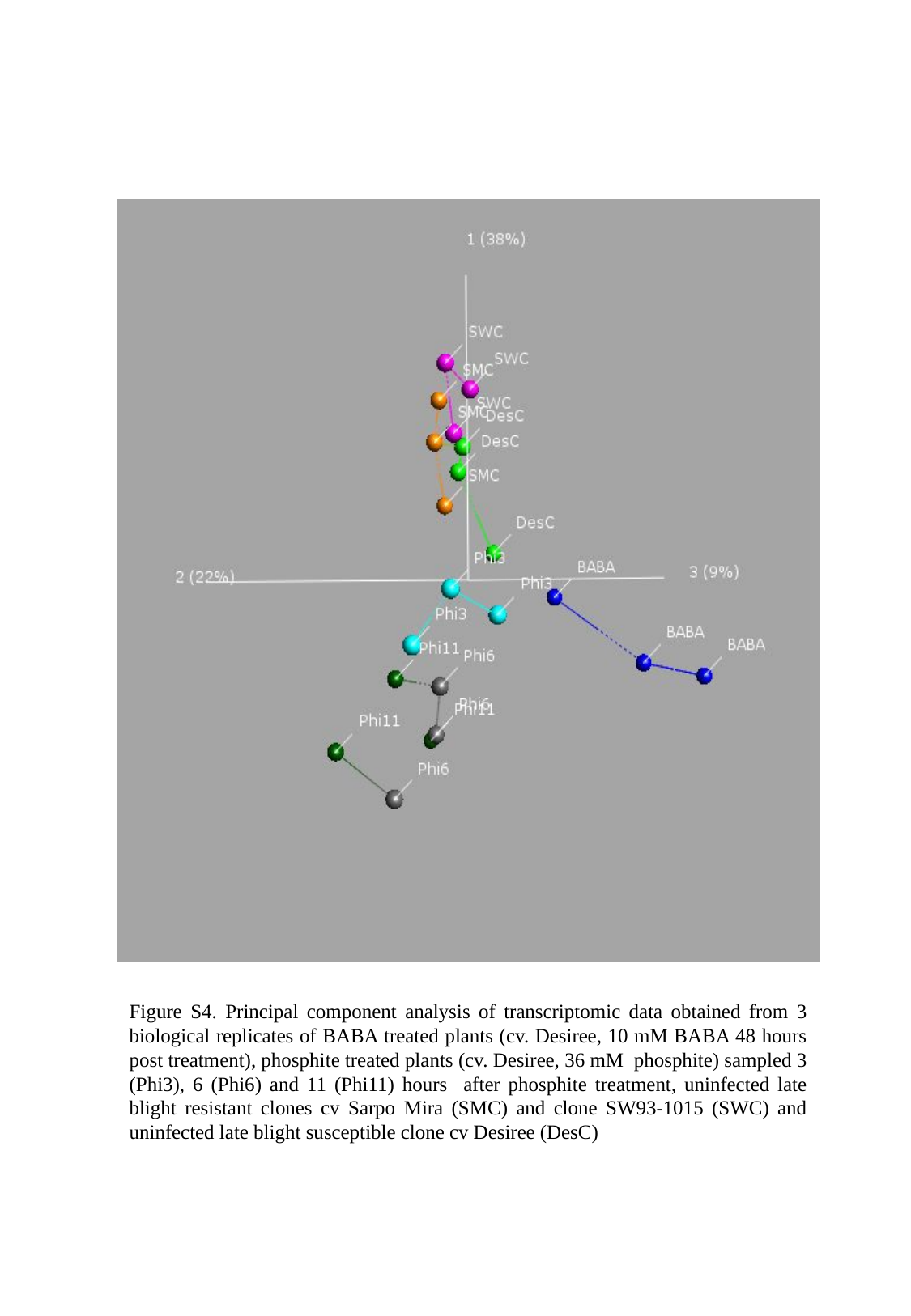

Supplement: Additional file 6: Figure S4. — Principal component analysis of transcriptomic data obtained from 3 biological replicates of BABA treated plants (cv. Desiree, 10 mM BABA 48 h post treatment) referred to as BABA, phosphite treated plants (cv. Desiree, 36 mM phosphite) sampled 3 (Phi3), 6 (Phi6) and 11 (Phi11) h after phosphite treatment, uninfected late blight resistant clones [58] cv Sarpo Mira (SMC) and clone SW93-1015 (SWC) and uninfected late blight susceptible clone cv. Desiree (DesC). [file 12870_2014_254_MOESM6_ESM.pptx]

## Slide 1
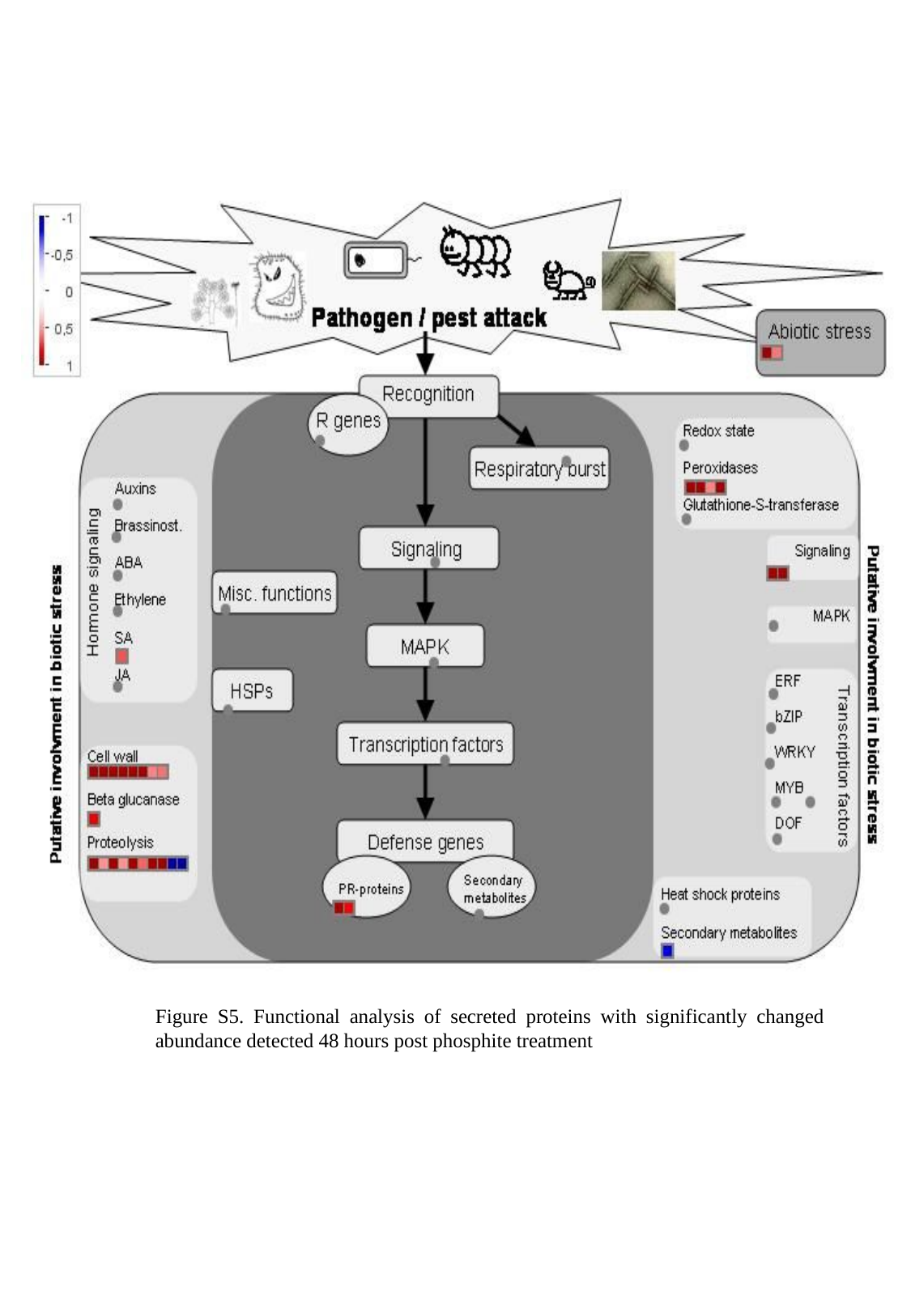

Supplement: Additional file 7: Figure S5. — Functional analysis using MapMan of secreted proteins with significantly changed abundance detected 48 h after phosphite treatment [77]. [file 12870_2014_254_MOESM7_ESM.pptx]

## Slide 1
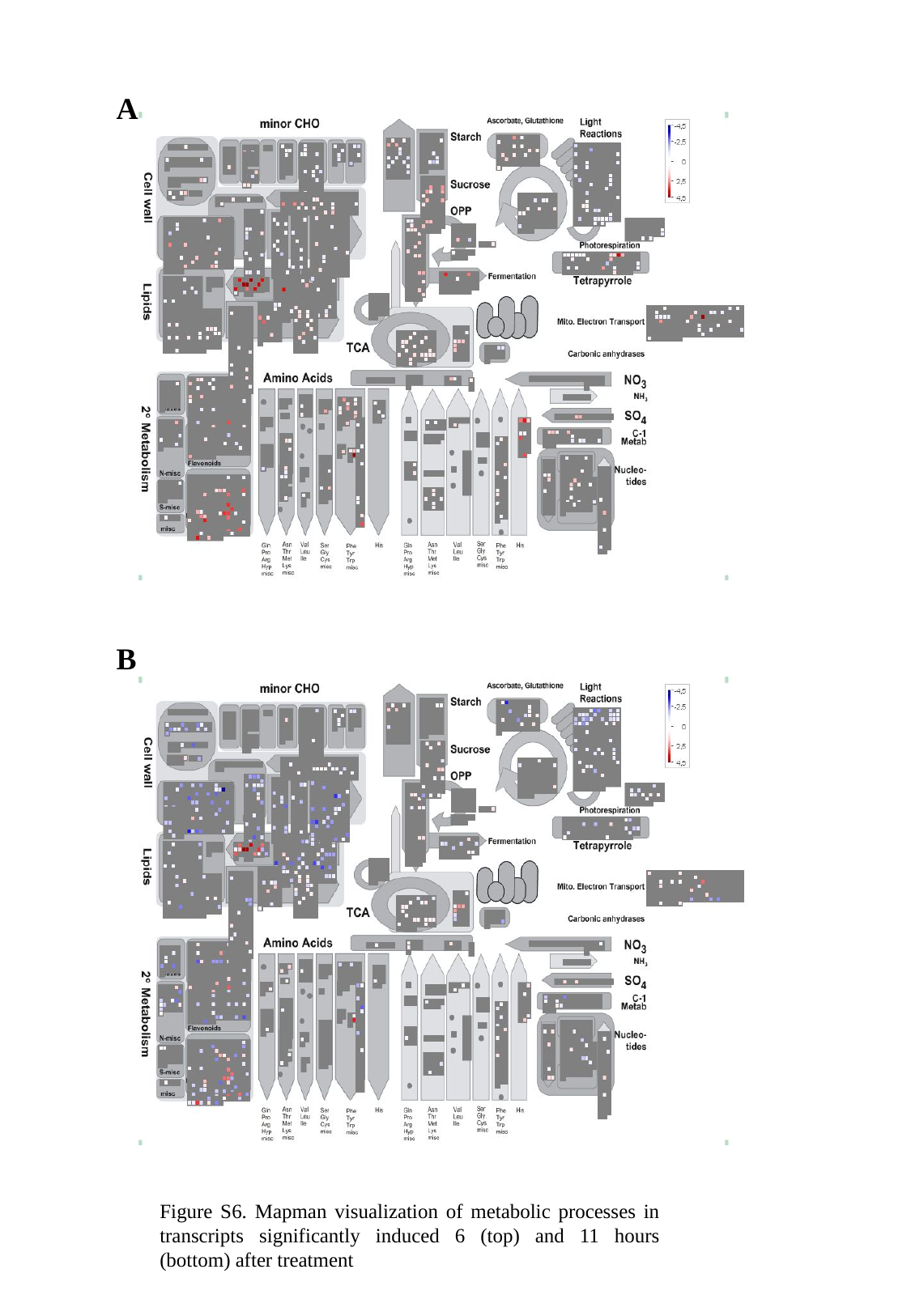

Supplement: Additional file 8: Figure S6. — Mapman visualization of metabolic processes in transcripts significantly changing 6 and 11 h after treatment. [file 12870_2014_254_MOESM8_ESM.pptx]
