# Supplementary material for: Phosphite-induced changes of the transcriptome and secretome in Solanum tuberosum leading to resistance against Phytophthora infestans
Source: BMC Plant Biol. 2014 Oct 1;14:254. doi: 10.1186/s12870-014-0254-y (PMC4192290; doi:10.1186/s12870-014-0254-y)
Supplement: Additional file 5: Table S2. — List of significantly enriched gene ontology terms among transcripts regulated both by BABA 48 h after treatment and phosphite 3 h after treatment. [file 12870_2014_254_MOESM5_ESM.docx]

Supplementary Table 2. List of significantly enriched gene ontology terms among transcripts regulated both by BABA 48 hours after treatment and phosphite three hours after treatment.

| GOID | Ontology | Term |
| --- | --- | --- |
| GO:0008150 | biological_process | biological_process |
| GO:0008152 | biological_process | metabolic process |
| GO:0009987 | biological_process | cellular process |
| GO:0044237 | biological_process | cellular metabolic process |
| GO:0044238 | biological_process | primary metabolic process |
| GO:0044281 | biological_process | small molecule metabolic process |
| GO:0044710 | biological_process | single-organism metabolic process |
| GO:0071704 | biological_process | organic substance metabolic process |
| GO:1901564 | biological_process | organonitrogen compound metabolic process |
| GO:0006464 | biological_process | cellular protein modification process |
| GO:0006468 | biological_process | protein phosphorylation |
| GO:0006793 | biological_process | phosphorus metabolic process |
| GO:0006796 | biological_process | phosphate-containing compound metabolic process |
| GO:0016310 | biological_process | phosphorylation |
| GO:0019538 | biological_process | protein metabolic process |
| GO:0036211 | biological_process | protein modification process |
| GO:0043170 | biological_process | macromolecule metabolic process |
| GO:0043412 | biological_process | macromolecule modification |
| GO:0044260 | biological_process | cellular macromolecule metabolic process |
| GO:0044267 | biological_process | cellular protein metabolic process |
| GO:0009058 | biological_process | biosynthetic process |
| GO:0009059 | biological_process | macromolecule biosynthetic process |
| GO:0034645 | biological_process | cellular macromolecule biosynthetic process |
| GO:0044249 | biological_process | cellular biosynthetic process |
| GO:1901135 | biological_process | carbohydrate derivative metabolic process |
| GO:1901137 | biological_process | carbohydrate derivative biosynthetic process |
| GO:1901576 | biological_process | organic substance biosynthetic process |
| GO:0031347 | biological_process | regulation of defense response |
| GO:0031348 | biological_process | negative regulation of defense response |
| GO:0048583 | biological_process | regulation of response to stimulus |
| GO:0048585 | biological_process | negative regulation of response to stimulus |
| GO:0080134 | biological_process | regulation of response to stress |
| GO:0006950 | biological_process | response to stress |
| GO:0006952 | biological_process | defense response |
| GO:0050896 | biological_process | response to stimulus |
| GO:0008219 | biological_process | cell death |
| GO:0012501 | biological_process | programmed cell death |
| GO:0016265 | biological_process | death |
| GO:0044699 | biological_process | single-organism process |
| GO:0044763 | biological_process | single-organism cellular process |
| GO:0006412 | biological_process | translation |
| GO:0006811 | biological_process | ion transport |
| GO:0006812 | biological_process | cation transport |
| GO:0044765 | biological_process | single-organism transport |
| GO:0000003 | biological_process | reproduction |
| GO:0007154 | biological_process | cell communication |
| GO:0008037 | biological_process | cell recognition |
| GO:0009856 | biological_process | pollination |
| GO:0009875 | biological_process | pollen-pistil interaction |
| GO:0022414 | biological_process | reproductive process |
| GO:0032501 | biological_process | multicellular organismal process |
| GO:0044703 | biological_process | multi-organism reproductive process |
| GO:0044706 | biological_process | multi-multicellular organism process |
| GO:0044707 | biological_process | single-multicellular organism process |
| GO:0048544 | biological_process | recognition of pollen |
| GO:0048610 | biological_process | cellular process involved in reproduction |
| GO:0051704 | biological_process | multi-organism process |
| GO:0006857 | biological_process | oligopeptide transport |
| GO:0015833 | biological_process | peptide transport |
| GO:0042886 | biological_process | amide transport |
| GO:0002376 | biological_process | immune system process |
| GO:0006955 | biological_process | immune response |
| GO:0055114 | biological_process | oxidation-reduction process |
| GO:0006497 | biological_process | protein lipidation |
| GO:0042157 | biological_process | lipoprotein metabolic process |
| GO:0042158 | biological_process | lipoprotein biosynthetic process |
| GO:0006725 | biological_process | cellular aromatic compound metabolic process |
| GO:0051716 | biological_process | cellular response to stimulus |
| GO:0009845 | biological_process | seed germination |
| GO:0090351 | biological_process | seedling development |
| GO:0007165 | biological_process | signal transduction |
| GO:0023052 | biological_process | signaling |
| GO:0044700 | biological_process | single organism signaling |
| GO:0006418 | biological_process | tRNA aminoacylation for protein translation |
| GO:0006436 | biological_process | tryptophanyl-tRNA aminoacylation |
| GO:0043038 | biological_process | amino acid activation |
| GO:0043039 | biological_process | tRNA aminoacylation |
| GO:0006979 | biological_process | response to oxidative stress |
| GO:0042221 | biological_process | response to chemical stimulus |
| GO:1901700 | biological_process | response to oxygen-containing compound |
| GO:0009607 | biological_process | response to biotic stimulus |
| GO:0009751 | biological_process | response to salicylic acid stimulus |
| GO:0010033 | biological_process | response to organic substance |
| GO:0014070 | biological_process | response to organic cyclic compound |
| GO:0051707 | biological_process | response to other organism |
| GO:0009719 | biological_process | response to endogenous stimulus |
| GO:0010200 | biological_process | response to chitin |
| GO:0010243 | biological_process | response to organic nitrogen |
| GO:1901698 | biological_process | response to nitrogen compound |
| GO:0010583 | biological_process | response to cyclopentenone |
| GO:0006928 | biological_process | cellular component movement |
| GO:0007017 | biological_process | microtubule-based process |
| GO:0007018 | biological_process | microtubule-based movement |
| GO:0006163 | biological_process | purine nucleotide metabolic process |
| GO:0006164 | biological_process | purine nucleotide biosynthetic process |
| GO:0006753 | biological_process | nucleoside phosphate metabolic process |
| GO:0006754 | biological_process | ATP biosynthetic process |
| GO:0006818 | biological_process | hydrogen transport |
| GO:0009116 | biological_process | nucleoside metabolic process |
| GO:0009117 | biological_process | nucleotide metabolic process |
| GO:0009119 | biological_process | ribonucleoside metabolic process |
| GO:0009141 | biological_process | nucleoside triphosphate metabolic process |
| GO:0009142 | biological_process | nucleoside triphosphate biosynthetic process |
| GO:0009144 | biological_process | purine nucleoside triphosphate metabolic process |
| GO:0009145 | biological_process | purine nucleoside triphosphate biosynthetic process |
| GO:0009150 | biological_process | purine ribonucleotide metabolic process |
| GO:0009152 | biological_process | purine ribonucleotide biosynthetic process |
| GO:0009163 | biological_process | nucleoside biosynthetic process |
| GO:0009165 | biological_process | nucleotide biosynthetic process |
| GO:0009199 | biological_process | ribonucleoside triphosphate metabolic process |
| GO:0009201 | biological_process | ribonucleoside triphosphate biosynthetic process |
| GO:0009205 | biological_process | purine ribonucleoside triphosphate metabolic process |
| GO:0009206 | biological_process | purine ribonucleoside triphosphate biosynthetic process |
| GO:0009259 | biological_process | ribonucleotide metabolic process |
| GO:0009260 | biological_process | ribonucleotide biosynthetic process |
| GO:0015672 | biological_process | monovalent inorganic cation transport |
| GO:0015985 | biological_process | energy coupled proton transport, down electrochemical gradient |
| GO:0015986 | biological_process | ATP synthesis coupled proton transport |
| GO:0015992 | biological_process | proton transport |
| GO:0018130 | biological_process | heterocycle biosynthetic process |
| GO:0019438 | biological_process | aromatic compound biosynthetic process |
| GO:0019637 | biological_process | organophosphate metabolic process |
| GO:0019693 | biological_process | ribose phosphate metabolic process |
| GO:0034220 | biological_process | ion transmembrane transport |
| GO:0034654 | biological_process | nucleobase-containing compound biosynthetic process |
| GO:0042278 | biological_process | purine nucleoside metabolic process |
| GO:0042451 | biological_process | purine nucleoside biosynthetic process |
| GO:0042455 | biological_process | ribonucleoside biosynthetic process |
| GO:0044271 | biological_process | cellular nitrogen compound biosynthetic process |
| GO:0046034 | biological_process | ATP metabolic process |
| GO:0046128 | biological_process | purine ribonucleoside metabolic process |
| GO:0046129 | biological_process | purine ribonucleoside biosynthetic process |
| GO:0046390 | biological_process | ribose phosphate biosynthetic process |
| GO:0055086 | biological_process | nucleobase-containing small molecule metabolic process |
| GO:0072521 | biological_process | purine-containing compound metabolic process |
| GO:0072522 | biological_process | purine-containing compound biosynthetic process |
| GO:0090407 | biological_process | organophosphate biosynthetic process |
| GO:1901293 | biological_process | nucleoside phosphate biosynthetic process |
| GO:1901362 | biological_process | organic cyclic compound biosynthetic process |
| GO:1901566 | biological_process | organonitrogen compound biosynthetic process |
| GO:1901657 | biological_process | glycosyl compound metabolic process |
| GO:1901659 | biological_process | glycosyl compound biosynthetic process |
| GO:0009620 | biological_process | response to fungus |
| GO:0050832 | biological_process | defense response to fungus |
| GO:0008610 | biological_process | lipid biosynthetic process |
| GO:0009611 | biological_process | response to wounding |
| GO:0010075 | biological_process | regulation of meristem growth |
| GO:0007166 | biological_process | cell surface receptor signaling pathway |
| GO:0007172 | biological_process | signal complex assembly |
| GO:0048832 | biological_process | specification of organ number |
| GO:0048833 | biological_process | specification of floral organ number |
| GO:0048443 | biological_process | stamen development |
| GO:0048466 | biological_process | androecium development |
| GO:0048653 | biological_process | anther development |
| GO:0051186 | biological_process | cofactor metabolic process |
| GO:0006778 | biological_process | porphyrin-containing compound metabolic process |
| GO:0006779 | biological_process | porphyrin-containing compound biosynthetic process |
| GO:0033013 | biological_process | tetrapyrrole metabolic process |
| GO:0033014 | biological_process | tetrapyrrole biosynthetic process |
| GO:0051188 | biological_process | cofactor biosynthetic process |
| GO:0006644 | biological_process | phospholipid metabolic process |
| GO:0008654 | biological_process | phospholipid biosynthetic process |
| GO:0006650 | biological_process | glycerophospholipid metabolic process |
| GO:0046486 | biological_process | glycerolipid metabolic process |
| GO:0006643 | biological_process | membrane lipid metabolic process |
| GO:0007568 | biological_process | aging |
| GO:0006505 | biological_process | GPI anchor metabolic process |
| GO:0006506 | biological_process | GPI anchor biosynthetic process |
| GO:0006661 | biological_process | phosphatidylinositol biosynthetic process |
| GO:0006664 | biological_process | glycolipid metabolic process |
| GO:0009247 | biological_process | glycolipid biosynthetic process |
| GO:0045017 | biological_process | glycerolipid biosynthetic process |
| GO:0046467 | biological_process | membrane lipid biosynthetic process |
| GO:0046474 | biological_process | glycerophospholipid biosynthetic process |
| GO:0046488 | biological_process | phosphatidylinositol metabolic process |
| GO:0005575 | cellular_component | cellular_component |
| GO:0016020 | cellular_component | membrane |
| GO:0005623 | cellular_component | cell |
| GO:0044464 | cellular_component | cell part |
| GO:0012505 | cellular_component | endomembrane system |
| GO:0016023 | cellular_component | cytoplasmic membrane-bounded vesicle |
| GO:0031410 | cellular_component | cytoplasmic vesicle |
| GO:0031982 | cellular_component | vesicle |
| GO:0031988 | cellular_component | membrane-bounded vesicle |
| GO:0071944 | cellular_component | cell periphery |
| GO:0044425 | cellular_component | membrane part |
| GO:0032991 | cellular_component | macromolecular complex |
| GO:0016469 | cellular_component | proton-transporting two-sector ATPase complex |
| GO:0033177 | cellular_component | proton-transporting two-sector ATPase complex, proton-transporting domain |
| GO:0008287 | cellular_component | protein serine/threonine phosphatase complex |
| GO:0005840 | cellular_component | ribosome |
| GO:0005789 | cellular_component | endoplasmic reticulum membrane |
| GO:0042175 | cellular_component | nuclear outer membrane-endoplasmic reticulum membrane network |
| GO:0044432 | cellular_component | endoplasmic reticulum part |
| GO:0000228 | cellular_component | nuclear chromosome |
| GO:0005578 | cellular_component | proteinaceous extracellular matrix |
| GO:0003674 | molecular_function | molecular_function |
| GO:0003824 | molecular_function | catalytic activity |
| GO:0005488 | molecular_function | binding |
| GO:0004672 | molecular_function | protein kinase activity |
| GO:0004674 | molecular_function | protein serine/threonine kinase activity |
| GO:0016301 | molecular_function | kinase activity |
| GO:0016740 | molecular_function | transferase activity |
| GO:0016772 | molecular_function | transferase activity, transferring phosphorus-containing groups |
| GO:0016773 | molecular_function | phosphotransferase activity, alcohol group as acceptor |
| GO:0016307 | molecular_function | phosphatidylinositol phosphate kinase activity |
| GO:0004702 | molecular_function | receptor signaling protein serine/threonine kinase activity |
| GO:0004871 | molecular_function | signal transducer activity |
| GO:0005057 | molecular_function | receptor signaling protein activity |
| GO:0008349 | molecular_function | MAP kinase kinase kinase kinase activity |
| GO:0060089 | molecular_function | molecular transducer activity |
| GO:0016538 | molecular_function | cyclin-dependent protein serine/threonine kinase regulator activity |
| GO:0019207 | molecular_function | kinase regulator activity |
| GO:0019887 | molecular_function | protein kinase regulator activity |
| GO:0030234 | molecular_function | enzyme regulator activity |
| GO:0004713 | molecular_function | protein tyrosine kinase activity |
| GO:0008047 | molecular_function | enzyme activator activity |
| GO:0019209 | molecular_function | kinase activator activity |
| GO:0030295 | molecular_function | protein kinase activator activity |
| GO:0042556 | molecular_function | eukaryotic elongation factor-2 kinase regulator activity |
| GO:0042557 | molecular_function | eukaryotic elongation factor-2 kinase activator activity |
| GO:0004872 | molecular_function | receptor activity |
| GO:0004888 | molecular_function | transmembrane signaling receptor activity |
| GO:0019199 | molecular_function | transmembrane receptor protein kinase activity |
| GO:0038023 | molecular_function | signaling receptor activity |
| GO:0035004 | molecular_function | phosphatidylinositol 3-kinase activity |
| GO:0019205 | molecular_function | nucleobase-containing compound kinase activity |
| GO:0051731 | molecular_function | polynucleotide 5'-hydroxyl-kinase activity |
| GO:0051735 | molecular_function | GTP-dependent polynucleotide kinase activity |
| GO:0004708 | molecular_function | MAP kinase kinase activity |
| GO:0004712 | molecular_function | protein serine/threonine/tyrosine kinase activity |
| GO:0008545 | molecular_function | JUN kinase kinase activity |
| GO:0004707 | molecular_function | MAP kinase activity |
| GO:0016909 | molecular_function | SAP kinase activity |
| GO:0008233 | molecular_function | peptidase activity |
| GO:0016787 | molecular_function | hydrolase activity |
| GO:0070011 | molecular_function | peptidase activity, acting on L-amino acid peptides |
| GO:0008443 | molecular_function | phosphofructokinase activity |
| GO:0019200 | molecular_function | carbohydrate kinase activity |
| GO:0004690 | molecular_function | cyclic nucleotide-dependent protein kinase activity |
| GO:0051734 | molecular_function | ATP-dependent polynucleotide kinase activity |
| GO:0004675 | molecular_function | transmembrane receptor protein serine/threonine kinase activity |
| GO:0005515 | molecular_function | protein binding |
| GO:0003676 | molecular_function | nucleic acid binding |
| GO:0097159 | molecular_function | organic cyclic compound binding |
| GO:1901363 | molecular_function | heterocyclic compound binding |
| GO:0004091 | molecular_function | carboxylesterase activity |
| GO:0016788 | molecular_function | hydrolase activity, acting on ester bonds |
| GO:0052689 | molecular_function | carboxylic ester hydrolase activity |
| GO:0016491 | molecular_function | oxidoreductase activity |
| GO:0005215 | molecular_function | transporter activity |
| GO:0000166 | molecular_function | nucleotide binding |
| GO:0001882 | molecular_function | nucleoside binding |
| GO:0001883 | molecular_function | purine nucleoside binding |
| GO:0005524 | molecular_function | ATP binding |
| GO:0017076 | molecular_function | purine nucleotide binding |
| GO:0030554 | molecular_function | adenyl nucleotide binding |
| GO:0032549 | molecular_function | ribonucleoside binding |
| GO:0032550 | molecular_function | purine ribonucleoside binding |
| GO:0032553 | molecular_function | ribonucleotide binding |
| GO:0032555 | molecular_function | purine ribonucleotide binding |
| GO:0032559 | molecular_function | adenyl ribonucleotide binding |
| GO:0035639 | molecular_function | purine ribonucleoside triphosphate binding |
| GO:0036094 | molecular_function | small molecule binding |
| GO:0043168 | molecular_function | anion binding |
| GO:1901265 | molecular_function | nucleoside phosphate binding |
| GO:0016817 | molecular_function | hydrolase activity, acting on acid anhydrides |
| GO:0008324 | molecular_function | cation transmembrane transporter activity |
| GO:0015075 | molecular_function | ion transmembrane transporter activity |
| GO:0022857 | molecular_function | transmembrane transporter activity |
| GO:0022891 | molecular_function | substrate-specific transmembrane transporter activity |
| GO:0022892 | molecular_function | substrate-specific transporter activity |
| GO:0004930 | molecular_function | G-protein coupled receptor activity |
| GO:0004812 | molecular_function | aminoacyl-tRNA ligase activity |
| GO:0016875 | molecular_function | ligase activity, forming carbon-oxygen bonds |
| GO:0016876 | molecular_function | ligase activity, forming aminoacyl-tRNA and related compounds |
| GO:0015077 | molecular_function | monovalent inorganic cation transmembrane transporter activity |
| GO:0015078 | molecular_function | hydrogen ion transmembrane transporter activity |
| GO:0022890 | molecular_function | inorganic cation transmembrane transporter activity |
| GO:0016298 | molecular_function | lipase activity |
| GO:0003777 | molecular_function | microtubule motor activity |
| GO:0004722 | molecular_function | protein serine/threonine phosphatase activity |
| GO:0005216 | molecular_function | ion channel activity |
| GO:0015267 | molecular_function | channel activity |
| GO:0022803 | molecular_function | passive transmembrane transporter activity |
| GO:0022838 | molecular_function | substrate-specific channel activity |
| GO:0022836 | molecular_function | gated channel activity |
| GO:0022839 | molecular_function | ion gated channel activity |
| GO:0015276 | molecular_function | ligand-gated ion channel activity |
| GO:0022834 | molecular_function | ligand-gated channel activity |
| GO:0004316 | molecular_function | 3-oxoacyl-[acyl-carrier-protein] reductase (NADPH) activity |
| GO:0004601 | molecular_function | peroxidase activity |
| GO:0016209 | molecular_function | antioxidant activity |
| GO:0016684 | molecular_function | oxidoreductase activity, acting on peroxide as acceptor |
| GO:0004177 | molecular_function | aminopeptidase activity |
| GO:0005261 | molecular_function | cation channel activity |
| GO:0008447 | molecular_function | L-ascorbate oxidase activity |
| GO:0016679 | molecular_function | oxidoreductase activity, acting on diphenols and related substances as donors |
| GO:0016682 | molecular_function | oxidoreductase activity, acting on diphenols and related substances as donors, oxygen as acceptor |
| GO:0004806 | molecular_function | triglyceride lipase activity |
| GO:0008484 | molecular_function | sulfuric ester hydrolase activity |
