# Supplementary material for: Phosphite-induced changes of the transcriptome and secretome in Solanum tuberosum leading to resistance against Phytophthora infestans
Source: BMC Plant Biol. 2014 Oct 1;14:254. doi: 10.1186/s12870-014-0254-y (PMC4192290; doi:10.1186/s12870-014-0254-y)
Supplement: Additional file 9: Table S3. — Significant differences observed in transcripts associated with salicylic and jasmonic acid pathways, the markers were selected as suggested in Studham, et al. [37]. [file 12870_2014_254_MOESM9_ESM.docx]

Supplementary Table 3. Significant differences observed in transcripts associated with salicylic and jasmonic acid pathways, the markers were selected as suggested in Studham et al. ([Studham and MacIntosh, 2012](#_ENREF_51)) (“- “ represents no significant expression observed)

**Jasmonic acid pathway**

| **ID** | **Function** | **Role** | **3 hour log2 fold change** | **6 hour log2 fold change** | **11 hour log2 fold change** |
| --- | --- | --- | --- | --- | --- |
| DMP400055325 | Linoleate 13S-lipoxygenase 2-1, chloroplastic | biosynthesis | - | - | 0.89 |
| DMP400029511 | Allene oxide synthase | biosynthesis | 1.93 | 1.81 | - |
| DMP400053803 | 12-oxophytodienoate reductase 3 | biosynthesis | - | 1.58 | - |
| DMP400051055 | AMP dependent CoA ligase | biosynthesis | - | 1.36 | 0.74 |
| DMP400035836 | Acyl-coenzyme A oxidase 2, peroxisomal | biosynthesis | - | 1.05 | 0.66 |
| DMP400018652 | Acyl-coenzyme A oxidase | biosynthesis | - | 0.78 | 1.07 |
| DMP400005281 | Jasmonate ZIM-domain protein 1 | signalling | 2.19 | 3.01 | 1.79 |
| DMP400050904 | Jasmonate ZIM-domain protein 3 | signalling | 1.2 | 1.49 | - |
| DMP400055226 | Jasmonate ZIM-domain protein 3 | signalling | - | - | 0.95 |
| DMP400005281 | Jasmonate ZIM-domain protein 1 | signalling | 2.19 | 3.01 | 1.79 |
| DMP400050864 | WRKY transcription factor 6 | signalling | - | 2.5 | - |
| DMP400016619 | Glutaredoxin | signalling | - | 2.23 | - |

**Salicylic acid pathway**

| **ID** | **Function** | **Role** | **3 hour log2 fold change** | **6 hour log2 fold change** | **11 hour log2 fold change** |
| --- | --- | --- | --- | --- | --- |
| DMP400037388 | Phenylalanine ammonia-lyase | biosynthesis | 3.73 | 3.51 | 1.29 |
| DMP400040591 | Phenylalanine ammonia-lyase | biosynthesis | - | - | -1.5 |
| DMP400037349 | Phenylalanine ammonia-lyase | biosynthesis | 1.86 | 1.06 | - |
| DMP400037388 | Phenylalanine ammonia-lyase | biosynthesis | 3.73 | 3.51 | 1.29 |
| DMP400041003 | BZIP transcription factor | signalling | - | 1.02 | 1 |
| DMP400055760 | Enhanced disease susceptibility 1 protein | signalling | - | 1.25 | - |
| DMP400034509 | Phytoalexin-deficient 4-2 protein | signalling | - | 1.56 | 1.51 |
| DMP400053604 | Acyl-[acyl-carrier-protein] desaturase | signalling | - | -1.2 | -1.5 |
| DMP400053601 | Acyl-[acyl-carrier-protein] desaturase | signalling | - | - | -1.3 |
| DMP400029302 | Double WRKY type transfactor | regulator | - | 2.3 | 1.16 |
| DMP400020631 | Double WRKY type transfactor | regulator | 3.19 | 3.71 | 2.39 |
| DMP400014725 | DNA-binding protein 4 | regulator | - | 1.33 | 1.75 |
| DMP400007677 | Ankyrin repeat-containing protein | signalling | - | 1.46 | 1.8 |
| DMP400012305 | SAG101 | signalling | 2.08 | 1.71 | - |
| DMP400055433 | NDR1 | signalling | 1.54 | 1.5 | 0.83 |
| DMP400044852 | Auxin and ethylene responsive GH3 | signalling |  | 1.53 |  |
| DMP400049274 | Transcription factor WRKY2 | signalling |  | 0.53 | 0.56 |
