# Supplementary material for: Phosphite-induced changes of the transcriptome and secretome in Solanum tuberosum leading to resistance against Phytophthora infestans
Source: BMC Plant Biol. 2014 Oct 1;14:254. doi: 10.1186/s12870-014-0254-y (PMC4192290; doi:10.1186/s12870-014-0254-y)
Supplement: Additional file 10: Table S4. — Primer sequences for qPCR. [file 12870_2014_254_MOESM10_ESM.docx]

| **Gene** | **PGSC (transcript)** | **For primer** | **Rev primer** |
| --- | --- | --- | --- |
| **StPEN1** | PGSC0003DMT400054964 | ACAACAGACGTCCTCGTCCT | ACGAGCTGAAAAACCTCGAA |
| **StAOS** | PGSC0003DMT400043495 | ACAACAGACGTCCTCGTCCT | ACGAGCTGAAAAACCTCGAA |
| **StFAD3** | PGSC0003DMT400021824 | GAATTGAACCCCCAGTAGCA | TCGATGGGTTCTCTGTTTCC |
| **StMLO1** | PGSC0003DMT400077489 | ATTGCGCGGACACTATAGGA | CTTGTGACGAATTAGGCCCG |
| **StWRKY8** | PGSC0003DMT400043203 | TCACCGACCTTCTTGCTTCT | ACTCCCTGTTGTCGGAGATG |
| **StLX-3** | PGSC0003DMT400028157 | ATTCCCTCCAAAAAGCCAGT | GGCTTCATTGTTCCATCGTC |
| **StWRKY1** | PGSC0003DMT400056352 | AATGGCAAGCATGGAAACTC | CACGTTACATCCTTGATGTGTG |
| **StNOD** | PGSC0003DMT400052060 | TGTGAAGGTGCCATATGCTT | CAATGCCAGCAAAACTCTGA |

Table S4: Primer sequences for qPCR
